# Characterizing uncertain sea-level rise projections to support investment decisions

**DOI:** 10.1371/journal.pone.0190641

**Published:** 2018-02-07

**Authors:** Ryan L. Sriver, Robert J. Lempert, Per Wikman-Svahn, Klaus Keller

**Affiliations:** 1 Department of Atmospheric Sciences, University of Illinois at Urbana-Champaign, Urbana, Illinois, United States of America; 2 RAND Corporation, Santa Monica, California, United States of America; 3 Earth and Environmental Systems Institute, The Pennsylvania State University, University Park, Pennsylvania, United States of America; 4 Department of Geosciences, The Pennsylvania State University, University Park, Pennsylvania, United States of America; 5 Department of Engineering and Public Policy, Carnegie Mellon University, Pittsburgh, Pennsylvania, United States of America; University of Miami School of Medicine, UNITED STATES

## Abstract

Many institutions worldwide are considering how to include uncertainty about future changes in sea-levels and storm surges into their investment decisions regarding large capital infrastructures. Here we examine how to characterize deeply uncertain climate change projections to support such decisions using Robust Decision Making analysis. We address questions regarding how to confront the potential for future changes in low probability but large impact flooding events due to changes in sea-levels and storm surges. Such extreme events can affect investments in infrastructure but have proved difficult to consider in such decisions because of the deep uncertainty surrounding them. This study utilizes Robust Decision Making methods to address two questions applied to investment decisions at the Port of Los Angeles: (1) Under what future conditions would a Port of Los Angeles decision to harden its facilities against extreme flood scenarios at the next upgrade pass a cost-benefit test, and (2) Do sea-level rise projections and other information suggest such conditions are sufficiently likely to justify such an investment? We also compare and contrast the Robust Decision Making methods with a full probabilistic analysis. These two analysis frameworks result in similar investment recommendations for different idealized future sea-level projections, but provide different information to decision makers and envision different types of engagement with stakeholders. In particular, the full probabilistic analysis begins by aggregating the best scientific information into a single set of joint probability distributions, while the Robust Decision Making analysis identifies scenarios where a decision to invest in near-term response to extreme sea-level rise passes a cost-benefit test, and then assembles scientific information of differing levels of confidence to help decision makers judge whether or not these scenarios are sufficiently likely to justify making such investments. Results highlight the highly-localized and context dependent nature of applying Robust Decision Making methods to inform investment decisions.

## 1. Introduction

Decision-makers planning for potential changes in future flood hazards grapple with the challenge of uncertain changes in future sea-levels and storm surges. One common approach to managing this uncertainty is based on defining deterministic scenarios of future changes in sea-levels [1,2] and choosing one or more scenarios, typically called the ‘best-estimate’, ‘worst case’ or ‘plausible upper bound’, as a basis for decision-making [3,4]. This approach, which we call the deterministic approach, has the advantage of being simple, but it has several drawbacks, particularly with regard to the potential for extreme sea-level rise because it provides little guidance on how to choose a worst case scenario nor how to incorporate that worst case into decision making [5,6]. The choice of a particular ‘plausible upper bound’ scenario is often subjective, but can have important consequences. For example, choosing a worst case scenario that is too lax might lead to overconfidence, while a too stringent choice could waste resources.

An alternative approach to manage uncertainty in sea-level changes is based on characterizing well-defined probability density functions and use this information in a risk-based decision framework, for example a cost-benefit analysis based on maximizing expected utility [4]. This approach, which we call the probabilistic approach, has the advantage of providing guidance on how to incorporate extreme cases into decisions, typically by weighing the impacts of such cases by their probability. However, the numbers provided by probabilistic approaches may not accurately represent the whole range of uncertain factors involved in predicting future sea-levels. In addition, most probabilistic projections of future sea-levels only present results conditional on different scenarios of future global emissions of greenhouse gases (the highest projections are typically recently based on the RCP8.5 scenario). The deep uncertainty of the many factors involved in assessing future sea-levels makes it difficult to construct with high confidence single probability estimates, which we discuss in more detail in the following section.

These problems can be managed within a probabilistic setting by also representing the (second order) uncertainty of the probability density function (also called ambigious or imprecise situations) [4]. An example of such an approach for coastal flood risk management is the sea-level rise allowances approach [7], which estimates a vertical height buffer that takes into account uncertainty not characterizable by a single probability density function using a convolution of probabilistic SLR projection with an extreme value distribution. Buchanan et al. [8] extends the sea-level rise allowances approach to include effects of ambiguity by also including users’ preferences regarding their confidence in sea level projections, time-horizon and risk tolerance. These sea-level rise allowances can help inform height adjustments that maintain under uncertainty annual expected probability of flooding, and it has been shown to be useful for local assessments [9].

Both the deterministic and the probabilistic approaches are designed to be incorporated into a common decision analytic process, one that first characterizes what is known about future states of the world and then uses this information to prescribe a best decision option (see section 2 for a deeper discussion of these approaches). In this paper, we present a third type of approach for characterizing uncertainty about future sea-level rise. This approach characterizes available information in relation to specific threshold values that would support, or not, a specific decision. As one advantage, such an approach provides a natural framework for incorporating information that spans a wide range of different types of uncertainty, from well characterized to deeply uncertain. Such an approach can be implemented using Robust Decision Making [10]. Robust Decision Making begins with a policy (or policies) under consideration and then identifies the combinations of physical and socio-economic factors that best distinguish futures in which the policy meets and misses its goals. Robust Decision Making makes it easier to explicitly distinguish among differing levels of scientific confidence in the relevant information. The approach is also designed to facilitate a “deliberation with analysis” process of decision support that helps parties to the decision reach consensus on actions they can take even if they do not agree on expectations about the future [11].

Previous Robust Decision Making analyses have used deeply uncertain climate information [12–14]. Here we explicitly use climate information of differing levels of uncertainty in the case of adapting to changes in sea-level and future storm surges. For example, we characterize the level of uncertainty for different contributions (e.g. thermal expansion, melting land ice, and dynamic effects due to changes in ocean surface topography) based on our understanding of the physical processes involved and the ability to model their behavior. This study demonstrates three important elements of the approach of how to: (1) use climate information with different levels of uncertainty, (2) combine uncertain climate information with uncertain information about relevant socioeconomic factors, and (3) display the results to decision makers.

Section 2 provides a background on assessing more extreme scenarios of future sea-level rise, and it also introduces the concept of ‘deep uncertainty’ and how Robust Decision Making can help manage deep uncertainty. Section 3 demonstrates our approach using a case study focused on the Port of Los Angeles. Results are provided in Section 4. Section 5 compares the results to a full probabilistic risk analysis and Section 6 provides a concluding discussion.

## 2. Background

### 2.1. Extreme scenarios of future sea-level rise

The IPCC Fifth Assessment Report (IPCC AR5) [15] reports a ‘likely’ sea-level rise range between 0.52–0.98 m by 2100 (as a global mean relative to 1986–2005) for the RCP8.5 scenario. The authors of the IPCC sea-level chapter have stressed that ‘likely’ should here be interpreted as “roughly a one-third probability that sea-level rise by 2100 may lie outside the ‘likely’ range” [16]. However, applications that require a high safety-level and for which future sea-levels are critical might also want to assess more extreme projections, which is the focus of the present paper. The uncertainty of the more extreme scenarios of future sea-level rise becomes a problem for both the deterministic and probabilistic approaches discussed above.

**Fig 1:**
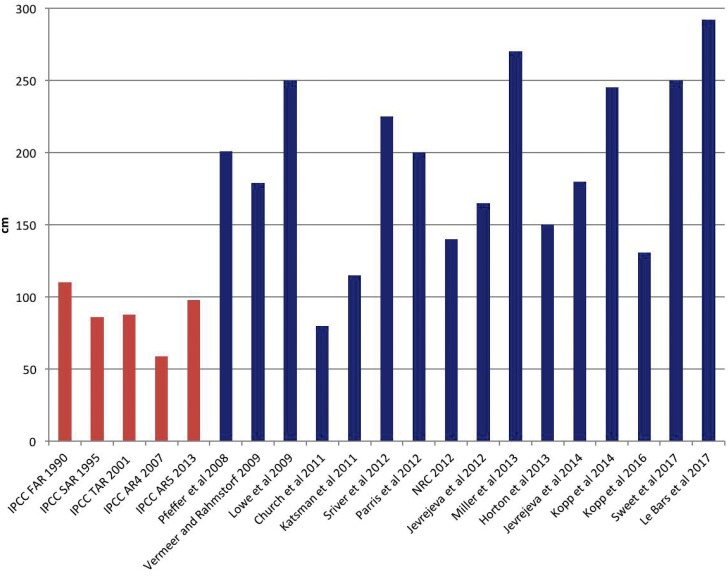
The highest projection or scenario of global mean sea-level rise (GMSLR) for the year 2100 for the five IPCC reports (red bars) and other key studies published after IPCC AR4 (blue bars). Note that that these numbers are not strictly comparable, as they are based on different assumptions regarding for instance emission scenarios, characterization of uncertainty (e.g. probability of exceedence) and reference years. See S1 Table for more details and sources to the numbers.

A main problem for the deterministic approach is that it is it is extremely difficult and contentious to determine what is the worst case scenario for future sea-level rise. The highest projections or scenarios of sea-level rise in 2100 documented in previous assessments have varied greatly (Fig 1 and S1 Table), which illustrates the difficulty in constraining the projected upper bound.

Determining a worst case scenario for future sea-level rise requires taking into consideration at least three main sources of uncertainty. The first is that most sea-level projections are conditional on scenarios of external factors (e.g., emissions of greenhouse gases/radiative forcings/temperature). For example, the choice of emission scenario can often dominate the outcome. It is therefore common to choose the upper range of the projections (based on the highest emission scenario) as a basis for the potential worst-case scenario. However, the emission scenarios commonly used for these projections do not represent absolute bounds [17]. If the range of emission scenarios does not span the full uncertainty range it can lead to overconfidence in the worst case scenario. The second source of uncertainty is due to the treatment of parameter uncertainty and internal variability, which can have large effects on the resulting projections [5,18]. A third source of uncertainty is the underlying model structures that are used. For example, there is ongoing scientific controversy over the reliability and robustness of so-called ‘semi-empirical models’ versus ‘process-based methods’ as a basis for making projections of future sea-level rise [19–21], in particular due to potential future acceleration of melting land ice [22–30]. However, our present understanding of the physical mechanisms contributing to rapid changes in the dynamics of ice flow is still limited [31,32]. For example, recent research shows how incorporating new structural mechanisms into ice sheet models can radically change the results [29,30,33,34]. This means that choosing a worst-case scenario for future sea-level rise is at this time far from trivial and uncontroversial. Constructing sea-level projections requires strategic choices, for example about emissions scenarios, consideration of sea-level contributions (e.g. thermal expansion, melting land ice, changes in ocean topography), and model structure. These choices can all influence results and interpretations in ways that may not be readily apparent to end-users of this information.

Characterizing sea-level projections in terms of probabilities goes back at least to Titus and Narayanan [35], who constructed a probability distribution of future sea-level rise informed by subjective expert assessments. More recently, Purvis et al. [36] inferred a probability distribution of sea-level rise based on the projections of the IPCC Third Assessment Report. Jevrejeva et al. [37] constructed a probability density function of global sea-level at 2100 by combining simulation experiments with results from formal expert elicitation. Kopp et al. [38] provided a full probability distribution for both regional and global sea-level change that has since been used in recent prominent North American studies [39,40]. Other recent studies also produce probabilistic sea-level rise projections and characterize aspects of the surrounding deep uncertainties [29,30,41,42].

The probabilistic approach also has to deal with the complexities and uncertainties of projecting future sea-level rise. Subjective probabilistic expert assessments can provide valuable information when appropriate experts are available, proper elicitation procedures are used, and when the experts’ judgments can be calibrated against known data [43,44]. Such conditions can however be difficult to fulfill when assessing never-before-observed phenomena driven by poorly understood processes. Evidence suggests that experts tend to be over confident when making judgments in the presence of uncertainty, with historical examples including numerical estimates of the speed of light, the mass of the electron and Avogadro’s number [45]. Perhaps not surprisingly, assessments about future sea-level rise have diverged in recent decades due to advances in modeling and understanding of the physical processes, an effect referred to as “negative learning” [46]. Le Cozannet et al. [47] argue that subjective expert knowledge is compatible with too many different probability functions, and that extra-probabilistic approaches are better suited to address the uncertainty in future sea level rise.

### 2.2. Characterizing uncertainty in future sea-level rise

Sea-level rise assessments must consider incomplete scientific knowledge, different methodological possibilities, and/or subjective judgments and interpretations about the analyses. Decision-making based on such information can be therefore seen as occurring under ‘deep uncertainty’. Lempert et al. [48] defines deep uncertainty as “the condition in which analysts do not know or the parties to a decision cannot agree upon (1) the appropriate models to describe interactions among a system’s variables, (2) the probability distributions to represent uncertainty about key parameters in the models, and/or (3) how to value the desirability of alternative outcomes.” (p 3–4) (see also Walker et al. [49]).

One implication of deep uncertainty is that a single probability distribution cannot fully capture all relevant information we have of the situation, in particular the degree of confidence that we have in the assigning exact probabilities to different outcomes. The use of uncertain probabilistic beliefs therefore presents challenges for standard approaches to decision making, such as expected utility maximization [50,51]. This raises questions on how much we should rely on the results based on probabilistic approaches. The problem is that practical decision-making based on a single probabilistic characterization of deeply uncertain parameters, such as a probability distribution of future sea-level rise, may be very sensitive to low-confidence information. It is therefore highly valuable if other approaches can be used to test the results of a decision analysis.

Both the deterministic and the probabilistic approaches discussed above can be seen as working within the normal paradigm of science-based decision-making, which have been called “science-first” [52], “predict-then-act” [53], and “agree on assumptions” [54] approaches. While scenarios of future sea-level rise can certainly be very useful for creating impacts assessments [55–57], the deterministic scenario approach can prove less useful for specific adaptation decisions, because it provides only very limited avenues to ensure that the scenarios being considered are those most relevant to the decisions that need to be made. A probabilistic characterization of future sea-levels may prove, as we have argued above, very difficult and contentious because well-characterized distributions for climate variables are not available and probabilistic projections of relevant socioeconomic factors may be even more unreliable [46,58–62].

Under conditions of deep uncertainty, it may prove more effective to begin with the specific decisions under consideration, and then use requirements associated with these decisions to identify the most important climate and socioeconomic scenarios to distinguish and consider. The literature offers several names for such approaches, including “context-first” [63], “assess risk of policy” [52,53,64], “vulnerability and robust response” [12], and “agree on decisions” [54]. These specific approaches differ in their characterizations of uncertainty, specific decision criteria, and the information they provide to decision makers (see for instance, the comparison in Hall et al. [12]). But they all share the central idea of defining a proposed policy or policies; identifying vulnerabilities of that policy, defined as conditions where the policy fails to meet its goals; identifying potential policy responses to those vulnerabilities; and then organizing scenarios to help policy makers decide whether and when to adopt those responses. The Thames River barrier plan provides an important example of such an approach in the context of sea-level rise [65,66]. Brown and Wilby [67] employ such approaches in water supply planning.

This paper focuses on a particular challenge in implementing such approaches—the question of how to organize a rich body of information about climate and socioeconomic factors into the scenarios that can be used to inform infrastructure investment decisions. Past applications have identified simple thresholds to represent their decision-critical scenarios, such as in the Thames River Barrier work. But in general, such scenarios will be multi-faceted, combining a range of different climate and other factors.

To address this challenge, the Robust Decision Making approach used in this study organizes the decision analysis around two questions: (1) under what future conditions would Port of Los Angeles (LA) find it advantageous to have hardened its terminal at the next upgrade, and (2) does current science and other available information suggest that these conditions are sufficiently likely to justify a decision to harden at the next upgrade? As discussed in more detail below, this allows us to use information of different levels of confidence at different stages of the analysis. The decision model (in this study a simple benefit cost calculation) uses single probability density functions representing the well-characterized climate and socio-economic uncertainties. The model is run over an experimental design informed by those factors regarded as deeply uncertain. The resulting database of model runs can then be used to identify decision-relevant scenarios, that is, the future conditions in which the proposed infrastructure investment fails to pass a benefit-cost test, and the probability threshold that this scenario would need to exceed to justify the investment. This probability threshold can then be compared to any low confidence probabilistic information that may be available regarding the deeply uncertain factors.

The distinction between those factors regarded as deeply uncertain and those regarded as well-characterized depends on analysts’ and/or decision makers’ confidence in the probabilistic judgments about those factors. It is useful to note that mis-characterizing a well-characterized uncertainty as deep incurs a computational penalty, expanding both the number of model runs required and the complexity of the data analysis. Mis-characterizing a deep uncertainty as well-characterized may, however, lead to a policy response brittle to unexplored uncertainties.

## 3. Materials and methods

Our approach requires a focus on specific decision in order to characterize the uncertainty. We thus demonstrate the approach with an idealized case study focused on the Port of LA. Many jurisdictions worldwide have been or are considering how to include sea-level rise into investments and management of large infrastructure investments [65,68–73]. The Port of LA provides a convenient and interesting example for how to address the potential for presumably low probability but large impact levels of extreme sea-level rise in investment plans and decisions.

We demonstrate the proposed approach for characterizing uncertainty by applying and discussing the methods in each step of the case study. The process consists of constructing a model for the decision problem that relates actions to consequences (Section 3.1), as well as a model of which uncertainties to treat as well characterized and which to treat as deep (section 3.2). The model of the decision problem and uncertainties are then used as the basis for identifying scenarios where the proposed policy fails to meet its goals (Section 4.1), and assessing whether the identified critical scenarios are sufficiently likely to justify taking an alternative decision (Section 4.2).

### 3.1. A model of the decision challenge for Port of Los Angeles

The Port of LA is one of the largest container shipping facilities in the world. It owns many square miles of land, but its main capitol stock consists of twenty container ship terminals—large steel and concrete structures that serve as docking facilitates for large container ships, the foundations for the large moving cranes that load and unload these ships, and transportation hubs for the trucks and trains that carry goods inland. When an organization such as Port of LA is building new infrastructure or conducting major renovations of existing facilities, it may prove useful to consider future sea-level rise. The effect of sea-level rise on Port of LA’s decisions regarding its container ship terminals follows this pattern. This study was conducted in collaboration with technical staff from the Port of LA. Port of LA was chosen because it provided an interesting and convenient case study. During the course of the study, Port of LA staff provided information and data and provided feedback on the analysis and results in a series of meetings.

**Fig 2:**
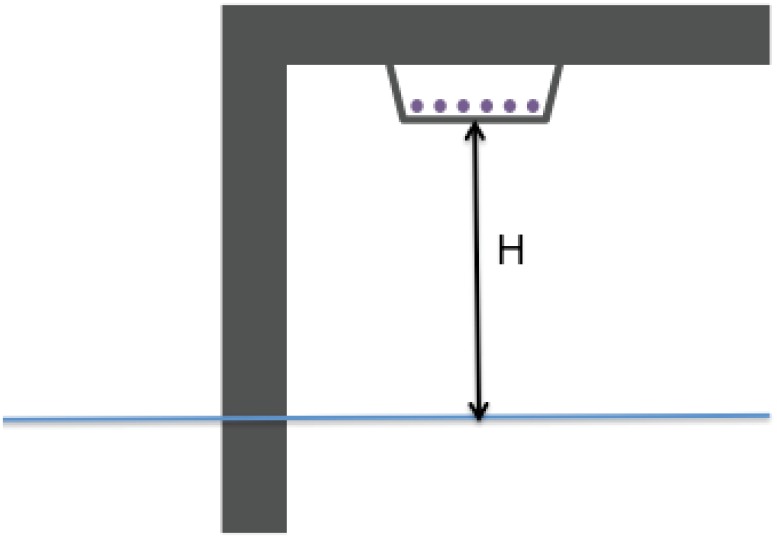
Schematic of Port of LA container ship terminal showing height (H) above mean sea-level.

The edge of Port of LA’s terminals currently lies about 3.7 meters above mean sea-level. As illustrated in Fig 2, conduits carrying high-voltage electric lines run underneath the main floor and lie 2.8 meters above mean sea-level. A breakwater, managed by the U.S. Army Corps of Engineers, provides the main barrier to wave action and storm surge in the harbor. The design and use of the terminals is driven by container ship technology. Port of LA first built such terminals in the 1960s and gave them a major overhaul in the 1980s when the size of container ships increased considerably. Several factors will drive the lifetime of Port of LA’s current terminals, including how long they take to wear out and any impending changes in container ship technology, both of which are uncertain. Port of LA’s terminals are relatively high above today’s mean sea-level and have never been flooded in the past few decades. The largest sea-level anomaly recorded at Port of LA from tide gauges during the past 100 years is roughly 1.5 meters, which is well below the current level of the conduits carrying the high voltage wires (3.7 meters above mean sea-level). Given this large apparent safety margin, Port of LA would only consider sea-level rise when planning a major upgrade of its terminals because hardening at that time would cost much less than it would at any other time.

**Fig 3:**
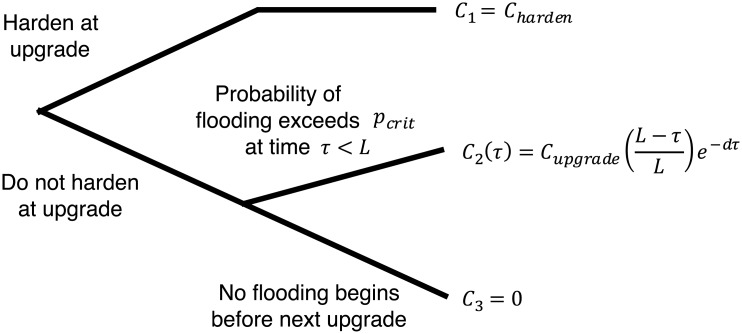
Simplified representation of Port of LA’s decision regarding whether or not to harden its terminal at its next upgrade and the costs resulting from its choices.

Using the Robust Decision Making approach described in Section 2.2, we approximate the Port of LA’s decision challenge as a sequential decision problem with a simple benefit cost framework. As shown in Fig 3, at some time in the future Port of LA will upgrade one of its terminals. It can decide to spend an additional sum *C*_*harden*_ to make the terminal practically invulnerable to plausible future sea-levels during the terminal lifetime. Such hardening might involve redesigning the electric conduits currently under the terminal and raising the terminal considerably higher. If the Port of LA decides to harden the terminal, they pay an additional *C*_*1*_ *= C*_*harden*_ now, and then suffer no further costs from any plausible amount of sea-level rise through the next upgrade, which would occur no less than several decades later. If the Port of LA decides not to harden its terminals at the next upgrade, and there is no sea-level rise large enough to flood the terminals, the costs are *C*_*3*_ *= 0*. However, if Port of LA decides not to harden, the terminal may prove vulnerable during its lifetime to sea-level rise. Storm surges combined with high tides and a higher mean sea-level might then occasionally flood the terminal. Such flooding would cause damage and disrupt operations.

We assume that Port of LA could tolerate some small flooding frequency, but if the flooding became too frequent, the organization would need to respond at significant cost. We assume that if the frequency of flooding exceeds this critical level (*P*_*crit*_), that Port of LA, rather than just hardening the existing terminal, would choose to conduct a major upgrade that would include the hardening. Note that this assumption may underestimate Port of LA’s future options, but it provides a simple representation of the current cost implications. A discussion of the implications of this and other assumptions is given below. If it chooses not to harden at the time of the next upgrade, they might be forced to upgrade in the future earlier than would otherwise be necessary. We assume that the main economic consequence of such an early upgrade is the early retirement of otherwise valuable infrastructure. Assuming the value of the terminal decreases linearly over its lifetime (i.e. linear depreciation), the resulting costs are

$$C_{2}(\tau )\;=\;C_{upgrade}(\frac{L-\tau }{L})e^{-d\tau },$$ (1)

where *C*_*upgrade*_ is the cost of the upgrade, *L* is the lifetime of the terminal in years, *d* is the discount rate in percent per year, and τ is the year when the frequency of flooding first exceeds the allowable threshold. The terminal lifetime is deeply uncertain because it depends on both the physical lifetime of the structure as well changes in shipping technology, which may or may not drive early obsolescence. Table 1 summarizes these and the other model parameters.

**Table 1:** Parameters affecting Port of LA’s decision whether or not to harden terminals at next upgrade and the treatment of the uncertainty in those parameters. Height and hardening cost values for a decision regarding PoLA terminals is discussed in Sections 3 and 4.

	Robust Decision Making Uncertainty Characterization	Full Probabilistic Uncertainty Characterization
**Future Sea-level**	Well-characterized joint probability distribution for a, b, and c.	Well-characterized joint probability distribution for a, b, c, c*, and t*.
Sea-level rise in 2011 (a)		
Normal rate of sea-level rise (b)		
Normal sea-level rise acceleration (c)		
Rate of abrupt sea-level rise (c*)	**Deeply uncertain** with range: 0 to 30 mm/yr	
Year abrupt rise begins (t*)	**Deeply uncertain** with range: 2010 to 2100	
Daily anomaly location (μ)	**Deeply uncertain** set of GEV distributions, with scale ranging from ψ = 517 to 569 mm, constant shape ξ = -0.305, and location μ = -176+0.1033(ψ-517) mm (constant mean).	Set of GEV distributions with constant shape ξ = -0.305, uniform distribution over scale 517 mm ≤ψ≤543mm, and corresponding location μ = -176 mm+0.1033(ψ-517mm)
Daily anomaly scale (ψ)		
Daily anomaly shape (ξ)		
**Future Terminal Management**		
Lifetime (L)	**Deeply uncertain** with range: 30 to 100 years	Consider a range of 30 to 100 years
Max allowable overtop probability (*P*_*crit*_)	**Deeply uncertain** with range: 5% to 50% per year	Consider uniform distribution over range 5% to 50% per year
Decision Year	Known at decision time: 2020	Known at decision time: 2020
Height (H) above mean sea-level	Known at decision time: 2804 mm	Known at decision time: 2804 mm
Current hardening cost (*C*_*Harden*_*/ C*_*upgrade*_)	Known at decision time: 1%	Known at decision time: 1%
Discount rate (d)	Known at decision time: 5%	Known at decision time: 5%

A decision to harden at the next upgrade would pass an economic cost-benefit test if the cost for doing so is less than the expected present value cost of any future early upgrade forced by sea-level rise. In other words, the expected present value of the savings due to the hardening (*S*_*Harden*_) should be positive. For convenience, we normalize all the costs to fractions of the upgrade cost so that *S*_*Harden*_ is approximated by the following expression:

$$S_{Harden}\;=\;\{\begin{array}{l} \text{exp}\;\left(-d\tau \right)\left(\frac{L-\tau }{L}\right)-\frac{C_{harden}}{C_{upgrade}}\;for\;\tau <L\\ -\frac{C_{harden}}{C_{upgrade}}\;for\;\tau \geq L \end{array}$$ (2)

The present value savings, and in particular the year τ, will depend on the future sea-levels, which is approximated as a sum of two time-series:

$$y_{t}\;=\;z_{t}+x_{t},$$ (3)

where z_t_ is the annual mean sea-level in the Port of LA for time index t, and x_t_ is the maximum hourly anomaly. We use an idealized time series estimator to approximate future mean annual sea-level as:

$$z_{t}\;=\;a+bt+ct^{2}+c^{*}I(t-t^{*}),$$ (4)

where the term a is the sea-level anomaly at time zero (2011), b is a constant rate (mm/year), and c is an acceleration term (mm/year^2^). (See Table 1 for a summary of the parameter definitions and symbols). To simplify our analysis, we assume that these first three terms represent only the effects of relatively well-understood processes, such as thermal expansion of the oceans due to rising temperatures and the melting of small glaciers, that are well-constrained by past observations. (As discussed in more detail in the next section, these terms should more properly be considered as a mix of well and less well understood processes.) The fourth term represents currently poorly understood and poorly constrained processes, for example, potentially abrupt changes in the dynamics of ice flow [34], which we estimate using a step-function increase in the rate of sea-level rise c* (mm/year) that occurs after some time t*.

While changes in the annual mean sea-level are an important driver, any actual flooding events will happen on much shorter timescales [71,74,75]. The local, hourly anomalies at Port of LA, x_t_, are well approximated by a generalized extreme value (GEV) distribution. Thus, we assume flooding would force an early upgrade in the first year τ in which:

$$P(x_{t}\geq H-z_{t})\;=\;1-exp\{-{\left[1+\xi \frac{H-z_{t}-\mu }{\psi }\right]}^{-1/\xi }\}\geq [1-{\left(1-p_{crit}\right)}^{\frac{1}{24*365}}],$$ (5)

where μ, ψ, and ξ are the GEV distribution’s location, scale, and shape parameters and the factor (24*365) translates the hourly frequencies into annual values.

We solve this model numerically by finding the smallest value of τ that satisfies Eq (5) and then evaluating the present value cost savings with Eq (2).

Any savings from a decision to harden at the next upgrade, as estimated by Eq (2), are contingent on the values of the fourteen parameters shown in Table 1. The calculation would be simple if these values were known precisely. The challenge is to evaluate the decision given large and divergent levels of uncertainty regarding these parameter values.

The Robust Decision Making approach addresses this challenge by answering two questions: (1) under what future conditions would Port of LA find it advantageous to have hardened its terminal at the next upgrade, and (2) does current science and other available information suggest that these conditions are sufficiently likely to justify a decision to harden at the next upgrade? We answer these questions through the following steps: 1) concisely summarizing the future conditions in which hardening at the next upgrade passes the cost-benefit test; 2) estimating the probability threshold that is the likelihood for these cases that would justify hardening at the next upgrade; and 3) evaluating scientific lines of evidence to help judge whether or not these cases are sufficiently likely to justify a decision to harden at the next upgrade. The following sections describe each of these steps.

### 3.2. Judging which uncertainties to treat as well characterized and which to treat as deep

To implement this Robust Decision Making analysis, we need to construct an experimental design that effectively samples over the plausible combinations of parameters in Table 1. The table divides the parameters affecting Port of LA’s potential savings into two categories: eight parameters describe future sea-level and six describe the terminal and its future management. The experimental design must appropriately combine parameters with different levels of uncertainty—some parameters have known values, some are best represented with well-characterized probability distributions, and some parameters are deeply uncertain. We categorize the uncertainties surrounding sea-level rise contributions as either well-characterizable or deeply uncertain based on several criteria, including our current understanding of the physical processes and capability of Earth system models to simulate observed trends and variability.

We treat four of the terminal management parameters—the decision year, height of the terminal, hardening cost, and discount rate—as known at the time of the decision. The other two terminal management parameters—the terminal lifetime and the maximum allowable annual flooding probability—refer to choices made by future Port of LA decision makers, and are thus deeply uncertain. We assume that the three coefficients of the quadratic expression for the well-understood processes of sea-level rise, the first three terms of Eq (4), can be accurately described with a single, well-characterized, joint probability distribution. We treat the other five parameters describing future sea-level rise—the rate and starting time of any abrupt changes and the three parameters describing the future distribution of hourly anomalies—as deeply uncertain.

**Fig 4:**
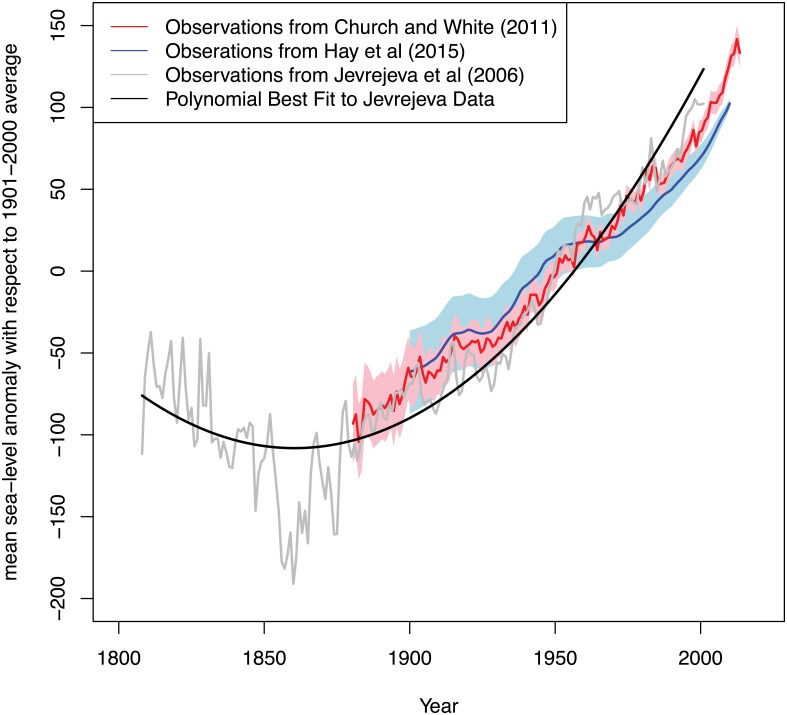
Observed annual global sea-level change based on Jevrejeva et al. [78] (gray curve), Hay et al. [81] (blue curve and shading), Church and White [76] (red curve and shading), and the polynomial best fit to the Jevrejeva et al. [78] data (black curve). Shading represents plus or minus one standard deviation.

We estimate a single, joint probability distribution over the parameters for what we have assumed to be the well-represented contributions to future sea-level rise by fitting the first three terms of Eq (4) to observed sea-levels over the past two centuries (Fig 4). The three polynomial parameters describe initial sea-level (corresponding to year ~2012), linear trend of sea-level rise, and non-linear (quadratic) sea-level acceleration. Similar to the many analyses that adopt sea-level rise projections based on simple, semi-empirical models or scenarios [36,70,71], we use a quadratic form that has provided useful insights [76–80] (Fig 4).

**Fig 5:**
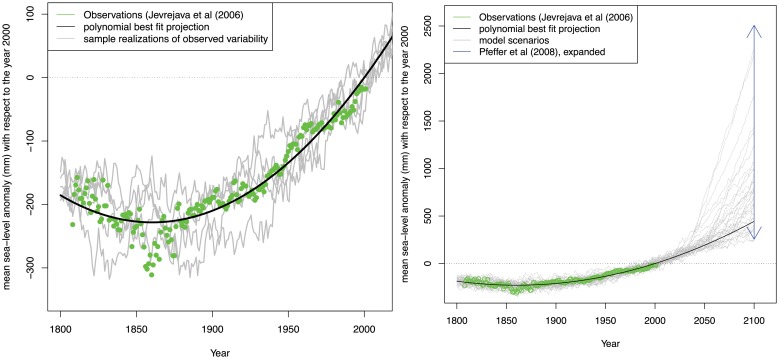
**Observed annually and globally averaged sea-level anomalies from Jevrejeva et al.** [78] **(green circles), the polynomial model best fit to the observations (black line) and model hindcast scenarios (grey lines) that sample the unresolved variability (left panel).** Bootstrap projections to 2100 highlighting potential future acceleration due to melting land ice (right panel). Projections are fitted to an idealized distribution of 2100 sea-level rise based on Pfeffer et al. [24] with an additional expansion to account for uncertainty in thermosteric sea-level rise [5,83].

The quadratic form provides a reasonably good fit to observed historical trends of global sea-level changes over the past 200 years. Projections of thermosteric sea-level rise using this semi-empirical approach generally agree with ranges shown in the IPCC Fourth and Fifth Assessments [15,82]. We use global sea-level rise from Jevrejeva et al. [78] as the basis for the polynomial fit, in order to be consistent with the original Port of LA study. It is meant as a proof-of-concept example highlighting the decision making methodology, and we note that newer observational SLR time series [38] and/or different time periods for the polynomial fitting can influence projected SLR ranges beyond what we consider here. The historical trends are generally consistent with more recent estimates [76,81] (Fig 4). For the projections, we combine the projected polynomial fit with the deeply uncertain future acceleration and resample the parameters to fit the idealized 2100 sea-level rise scenarios (Fig 5). Note the contributions of observational errors and differences in historical data sets to projected sea-level uncertainties (shown in Fig 4) are relatively small compared to the deeply uncertain polar land ice contributions for extreme sea-level rise scenarios (Fig 5).

We do not include regional assessments or probabilistic projections here, which can be useful for local studies [38–40,84]. We fit our time series projections to different theoeretical probability density functions of 2100 sea-level rise (described below), reflecting distinctly different attitudes and expectations about future sea-level rise including deep uncertainty surrounding the upper-bound. Using more complex sea-level models [29,85,86] would improve the physical realism of the analysis, but would also significantly complicate and, we hypothesize, not considerably affect the conclusions of the decision-analyses described below.

A simple bootstrap analysis accounting in an approximate way for auto-correlation in the model residuals [87] (discussed below) is used to estimate the joint distribution of the parameters a, b, and c using observations of globally and annually averaged sea-levels [78]. The sea-level observations are normalized to a zero anomaly in the year 2000 to simplify comparisons with other studies such as CO-CAT [88]. We fit the model in a least-squares sense, approximate the data-model residuals using an autoregressive model of order one, superimpose bootstrap realizations of the residuals to the original fit, and then re-estimate the parameters for each bootstrap realization. This process provides a distribution of a, b, and c that approximates past observed sea-level variability quite well, as well as future projections based on semi-empirical [89] and mechanistic ocean models [5]. We have conducted additional sensitivity tests to the auto-regressive estimator and our parameter estimates are generally robust to different values of innovation variance reflecting time variations in the observational error in sea-level.

Local observed and projected sea-level rise rates can differ substantially from global estimates. For high global mean sea-level rise futures, the regional response along the western coast of North America may be larger than the global average, due to the fingerprint effect of Antarctic ice mass loss [38]. We represent the projection uncertainties introduced by the discrepancies between local and globally averaged sea-levels by expanding the uncertainty range of future sea-level rise (discussed below). The use of the globally averaged data (as opposed to local observations) is an approximation and reflects the idealized nature of this sensitivity analysis. This approximation guards to some extent against the effects of the observed decadal-scale oscillations in the rate of regional sea-level rise and the resulting potential for a considerable increase in the rate of sea-level rise in the Eastern Pacific, for example due to circulation effects [78,90]. In addition, this approximation makes it easier to link the sea-level rise projections affected by well-represented uncertainties to studies analyzing deep uncertainties (discussed next).

The other uncertainties in Table 1—five for future sea-level and two for future terminal management—are deep. The deeply uncertain parameters include the rate (c*) and onset timing (t*) of abrupt sea-level rise associated with potential future accelerations due to melting land ice (see also Wong et al. [91] as well as Diaz and Keller [92]), as well as the location, scale, and shape parameters of the tail area distribution of sea-level anomalies accounting for potential future changes in the behavior of extremes. The deeply uncertain terminal management parameters include the lifetime and maximum allowable overtop probability. These uncertainties are represented by a range, or set, of plausible values. For each parameter a range is chosen that is consistent with physical or other constraints and sufficiently wide to contain the boundary between cases where hardening at the next upgrade does and does not meet the cost-benefit test.

Port of LA has no experience with extreme flooding and thus no solid estimates regarding the maximum allowable flooding probability—that is, the frequency of annual flooding that would force the organization to undertake an early terminal upgrade. We hence choose a wide range of values, between a 5 percent and 50 percent chance of annual flooding, that would force an early upgrade. The 5 percent criterion was provided by Port of LA. The probabilities are relatively high compared to other typical flood risk assessments (e.g. a one percent chance of flooding per year, see, for example, Jonkman et al. [93], which may reflect a concern with the costs of business interruption rather than that of physical damage to facilities themselves. Along these lines, we also choose a wide range for the lifetime L of its terminals to be between 30 and 100 years.

The parameters c* and t* represent the contribution of poorly understood processes to future annual mean sea-level rise. As described in the next section, we choose the values of c* and t* to approximate two expert assessments. For t*, we add as an additional constraint that such a rise could begin immediately, and we take as our upper bound the end of the considered time horizon (the year 2100), where a rapid rise would no longer be relevant to any near-term hardening decision by Port of LA.

**Fig 6:**
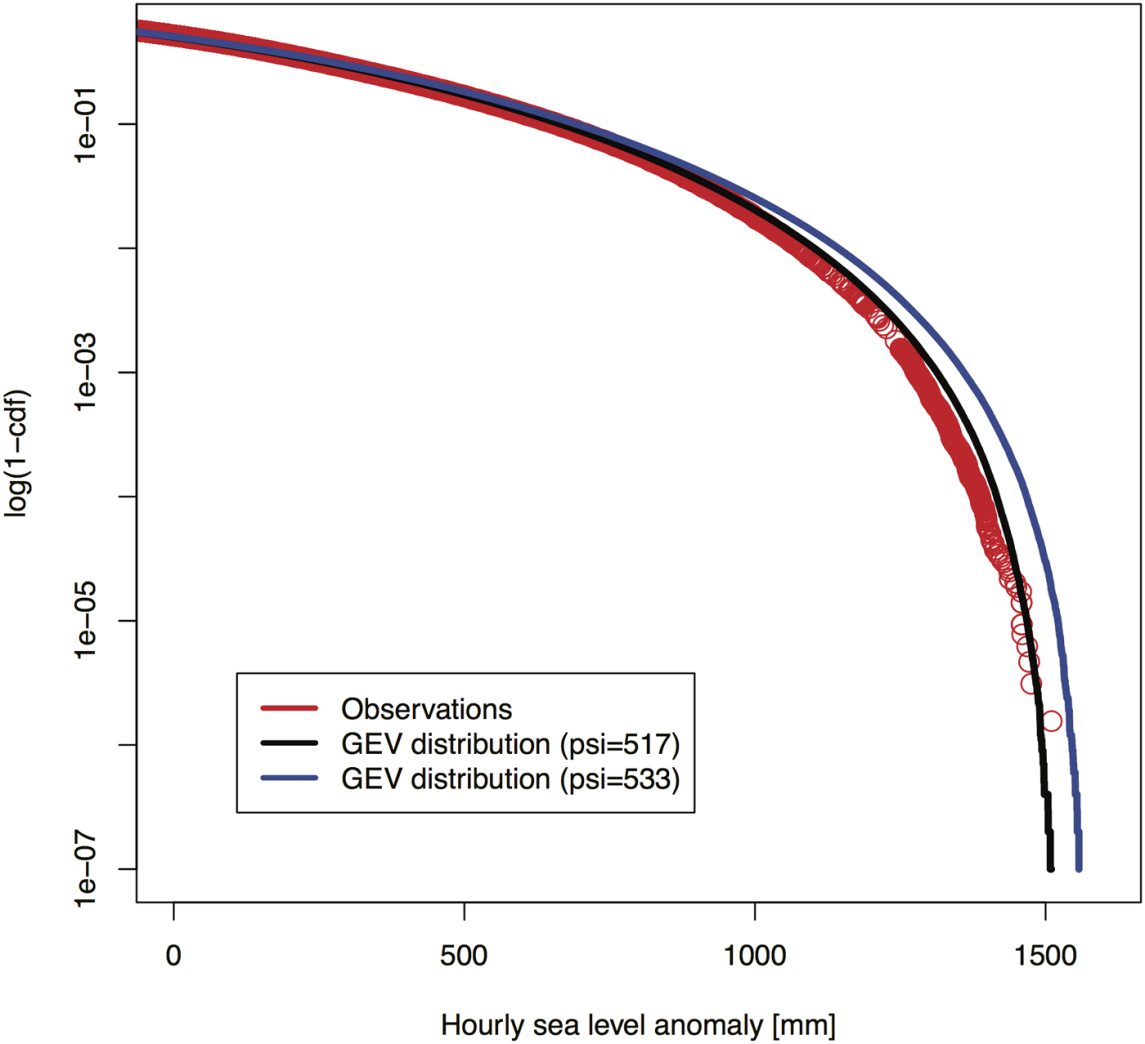
Black line shows the General Extreme Value (GEV) distribution fitted to the hourly sea-level anomalies from the annual mean values observed near the Port of LA [94]. The interpolated GEV distribution parameters are given in Table 1. The blue line shows the GEV distribution with an expanded scale parameter of y considered in the decision analysis described in the main text.

To treat the future daily anomaly, we begin with the common assumption of stationarity in the intra-annual variability, modeled by superimposing an estimate of the past variability on the projected future changes in the annual mean. The State of California Sea-level Rise Interim Guidance Document (CO-CAT [88]) assessed this as a “reasonable starting point” because little information exists to project any future changes in this variability. A time series of past anomalies at Port of LA is generated by subtracting the observed change in the annual mean from local observations [94] spanning roughly eight decades. A GEV distribution is then fit to these anomalies. We employ the GEV as a simple and parameterizable interpolator that yields good agreement with the observations. Note, we use the GEV fit here to highlight the model sensitivity, rather than for parameter estimation. As shown in Fig 6, a fit with location μ = -176 mm, scale ψ = 517 mm, and shape ξ = -0.305 reproduces the observed anomalies quite well. Note also that the hourly anomalies range over roughly 3000 mm, which is approximately two orders of magnitude larger than the standard deviation of the unresolved interannual variability of approximately 24 mm. As an approximation in the interest of model parsimony, we hence neglect the relatively small effects of the unresolved intra-annual variability for the projection of flooding risks.

There is, however, no guarantee that the future distribution of hourly anomalies will remain stationary due to climate change and other factors [95,96]. To represent these potential, deeply uncertain future changes we consider a set of GEV distributions, created by varying the scale parameter over the range 517 mm ≤ ψ ≤ 569 mm, where the lower bound is the current scale and the upper bound is 10 percent larger. In general, other distribution parameters (such as the shape parameter) could also be varied, but as shown below there is insufficient scientific information available to justify this degree of fidelity. The mean of a GEV distribution is given by the expression *μ*+*ψ*[Γ(1−*ξ*)−1]/*ξ*, where Γ() is the gamma function [97]. As the scale varies, the mean of the hourly anomaly around the annual mean must remain constant (our treatment of the anomalies demand that they do not shift the annual mean), so we write the location of each distribution in our set as

μ=-176-(ψ-517mm)[Γ(1.305)-1]=-176+0.1033(ψ-517mm) (6)

## 4. Results

### 4.1. Identifying scenarios where the policy fails to meet its goals

Our initial analysis considers a decision where Port of LA upgrades a terminal in 2020; the costs of hardening are small, C_harden_/C_upgrade_ = 1 percent; and Port of LA uses a discount rate of 5 percent per year. As this analysis will show, this low hardening cost is considered because it is at the high end of near-term investments Port of LA might reasonably make to protect its terminals against extreme sea-level rise.

We begin by evaluating the decision model using both the deeply uncertain and well-characterized uncertainties. We generate a 500-point Latin hypercube (LHC) sample [98] over the five deeply uncertain parameters: three for future sea-level rise (c*, t*, *ψ*) and two for future terminal management (*L* and *p*_*crit*_), using the parameter ranges shown in Table 1. The LHC method provides a numerically efficient sample of the space of deeply uncertain parameters. For each case in this sample, we calculate the expected cost using the parameters representing well-characterized uncertainty. In particular, we calculate the cost savings for 700 equally likely combinations of values for the parameters a, b, and c. Thus, for each of the 500 cases in the LHC sample, the expected savings of a decision to harden at the next upgrade is calculated, contingent on the distribution for the parameters with well-characterized uncertainty (a, b, and c) and on a particular set of values for the deeply uncertain parameters c*, t*, *ψ*, *L*, and *p*_*crit*_).

**Fig 7:**
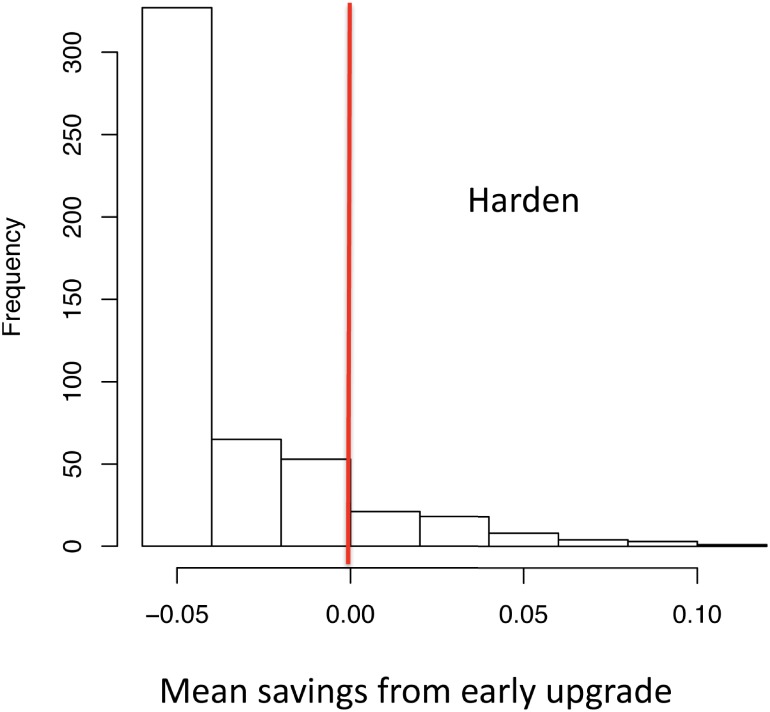
Histogram of model results generated with 500-point Latin Hypercube sample over deeply uncertain parameters in Table 1. Positive values indicate cases in which hardening at next upgrade passes a cost-benefit test.

The results of this economic analysis (Fig 7) show that in 327 of the 500 cases a decision to harden at the next upgrade would fail a cost benefit test. In 173 of the cases, such a decision would have some cost savings. In a small number of those cases, the cost savings would be quite large, up to 20 times the cost of hardening.

We next perform a scenario discovery analysis using this database of 500 cases to identify a scenario in where Port of LA might regret a decision not to harden at the next upgrade. Scenario discovery [99] applies a cluster analysis to a database of simulation model results, seeking to identify those combinations of uncertain input parameters, which most concisely predict certain policy-relevant outcomes. Here we seek those combinations of the five deeply uncertain parameters that best predict those cases where a decision to harden at the next upgrade would pass the cost-benefit test. Previous applications of scenario discovery have used PRIM (patient rule induction method) to identify these clusters of cases. PRIM is a user-interactive bump-hunting algorithm that identifies hyper-rectangular regions in the input space of the simulation model [100]. Here this approach is augmented by first applying a principle component analysis (PCA) to the parameters c* and t* and then applying PRIM to the resulting rotated set of parameters. The PCA-based preprocessing step transforms the original model input parameters so that PRIM can then identify high quality hyper-rectangular scenarios in the new rotated coordination system [100]. This PCA and PRIM combination can prove useful in situations where the scenarios can be best described by linear combinations of some uncertain input parameters, rather than just hyper-rectangular regions in the space of original input parameters.

This scenario discovery analysis suggests, as shown in Table 2, that the decision to harden at the next upgrade might pass the cost-benefit test in cases with a near-term and rapid increase in sea-level, given by $c^{*}\geq 14\frac{mm}{yr}+0.3\frac{mm}{yr}(t*-2010)$; a long terminal lifetime, given by L>50 years; and a significant increase in the hourly anomaly, given by ψ>533 mm. The value of the critical threshold *p*_*crit*_ appears relatively unimportant to Port of LA’s decision of whether or not to harden at the next upgrade.

**Table 2:** Parameter ranges defining the Harden at Next Upgrade scenario. The center column shows the conditions under which a decision to harden at the next upgrade would pass a cost-benefit test.

Parameter	Low	Pass cost-benefit test	High
$c^{*}-0.3\frac{mm}{yr}(t^{*}-2010)$	$\geq -27\frac{mm}{yr}$	$\geq \;14\frac{mm}{yr}$	$\geq \;30\frac{mm}{yr}$
*L*	≥30 years	≥50 years	≥100 years
*ψ*	≥517 mm	≥533 mm	≥569 mm

These three conditions define a cluster of cases that we label the Harden at Next Upgrade scenario. As described in Bryant and Lempert [99], this cluster can serve as a scenario useful for decision-making. The three conditions represent the scenario’s driving forces. The cluster analysis also provides two measures—coverage and density—of the scenario’s quality. This scenario has coverage of 63 percent, that is 109 of the 173 cases in the LHC sample where hardening at the next upgrade passes a cost-benefit test meet the three conditions in Table 2. The scenario has density of 96 percent, that is, of the 113 cases in the sample that satisfy the conditions shown in Table 2, 109 of them pass the cost-benefit test.

It is possible to calculate a probability threshold (*P*_*thres*_) for this scenario, that is, the likelihood Port of LA would have to ascribe to it so that the expected cost savings for hardening at the next upgrade are greater than zero. Note the probability threshold (*P*_*thres*_) is separate from the critical probability (*P*_*crit*_) defined previously representing the critical frequency of flooding that would lead to a major upgrade including hardening. This probability threshold *P*_*thres*_ is the smallest value that satisfies

$$P_{thres}{\overset{-}{S}}_{Harden\;Scenario}+(1-P_{thres}){\overset{-}{S}}_{All\;Other\;Cases\;}\geq 0.$$ (7)

where ${\overset{-}{S}}_{Harden\;Scenario}$ is the average savings of the cases that satisfy the conditions shown in Table 2 and ${\overset{-}{S}}_{All\;Other\;Cases}$ is the average savings of all the other cases in the considered parameter sample. We estimate these averages with a uniform distribution over the two respective sets of cases, which yields a *P*_*thres*_> 7%.

Thus Port of LA might reasonably chose to harden its terminals at the next upgrade if they judged the probability of the Harden at Next Upgrade scenario, as defined by the conditions in Table 2, to be at least 7 percent. Note that this represents a lower bound to the probability threshold, since the uniform distribution over the futures in the “Harden at the Next Upgrade” scenario weights the futures with high and low mean savings equally. If the futures on the far right-hand side of Fig 7 were less likely than those near the vertical red line, then the probability threshold would be higher, and the conclusions in Section 6 strengthened.

### 4.2. Assessing whether the identified policy-relevant scenarios are sufficiently likely to justify taking an alternative decision

We now analyze information that can help inform the judgments regarding the likelihood of the Harden at Next Upgrade scenario. In particular, climate science can help inform judgments about the likelihood of values of the parameters c*, t*, and ψ that satisfy the conditions that define this scenario.

**Fig 8:**
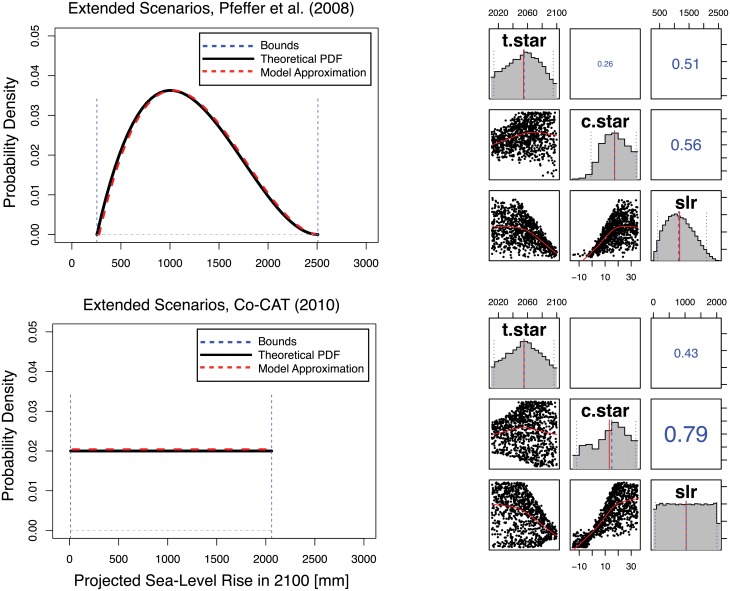
Parameter estimates resulting from the model calibration to the: (top) extended scenarios of Pfeffer et al. [24], and (bottom) the CO-CAT [88] scenarios. The former uses a beta distribution and the latter a uniform distribution, and both begin with a uniform prior, as described in the text.

Note first that the condition $c^{*}\geq 14\frac{mm}{yr}+0.3\frac{mm}{yr}(t*-2010)$ implies a sea-level rise contribution from poorly understood processes of about 1400 mm in 2100. When combined with the roughly 500 mm contribution from well-understood processes, the Harden at Next Upgrade scenario implies a roughly 2 meter (m) sea-level increase by century’s end. Such a level is within, but at the high end, of some current sea-level rise projections (see Figs 1 and 8). This suggests that the scenario may be less likely than the 7 percent threshold derived from the economic analysis. A more detailed understanding can result from estimates of joint probability distributions for c* and t*. While imprecise, such probability estimates can usefully contribute to judgments about the conditions under which Port of LA might consider hardening its terminals at the next upgrade.

Joint probability distributions are estimated for c* and t* by sampling from broad prior distributions and applying a rejection sampling to approximate the results of these bounding analyses. This study uses two different sets of projections—a modification of the analysis of Pfeffer et al. [24] and the California Sea-level Rise Interim Guidance document CO-CAT [88]—which yield two different joint probability distributions and thus a range of estimates for the likelihood of the condition $c^{*}\geq 14\frac{mm}{yr}+0.3\frac{mm}{yr}(t*-2010)$.

Pfeffer et al. [24] analyze kinematic constraints on the sea-level rise contributions from land-based ice and derive lower and upper bounds of 785 and 2008 mm for sea-level rise in the year 2100 and a “more plausible” estimate of about 800 mm. Note this range extends well beyond the “likely” upper bound of ~1 meter documented in the IPCC’s Fifth Assessment Report [15]. However, as documented in the Summary for Policy Makers, there is low confidence in the representation of ice sheet dynamics in the process-based models used to make these projections. Hence, scenarios with larger sea-level rise are possible, but deeply uncertain.

We introduce two adjustments to the Pfeffer et al. [24] results because these previous results neglect uncertainties due to thermosteric sea-level rise [5,83] and the divergence between global mean and local sea-level change. The lack of uncertainty assessment of about the thermosteric sea-level rise component is addressed by adding an additional rise of -230 to + 200 mm. This uncertainty range is derived from a comparison of observed sea-levels and an ensemble of runs from an Earth System Model of Intermediate Complexity, that includes a three-dimensional dynamic ocean general circulation model and samples key parametric uncertainties. The asymmetry of this range is due to the slight difference between the median thermosteric sea-level rise estimate adopted by Pfeffer et al. [24] and the estimate from Sriver et al. [5]. The local circulation effects are approximated with an additional rise of +/- 300 mm. This range is approximately the range of projected local sea-level rise anomalies with respect to the global mean at the end of this century [82]. This range is also roughly consistent with the divergence of the simple parabolic fit to the local (Port of LA) and global observations [78] extrapolated to the year 2100, but we note that they are relatively large compared to recent plausible range of +/- 14 cm, based on CMIP5 analysis [38]. These two adjustments yield modified lower and upper bounds for the annual mean local sea-level in 2100 of 255 mm to 2508 mm with a more plausible value of 950 mm (Fig 8). This line of evidence is then approximated using a rescaled beta distribution, chosen because it provides a good approximation of the upper and lower bounds, as well as the most-likely regions.

The California Sea-level Rise Interim Guidance Document (CO-CAT [88]) reviewed a number of published sea-level rise projections and derived a sea-level rise range between 310 and 1760 mm for California in the year 2100. Note that these “projections do not account for catastrophic ice-melting” and are for a specific region (as opposed to the global mean). In the same way as we have modified the Pfeffer et al. [24] scenario, we approximate the local circulation effects with an additional rise of +/- 300. This results in a modified CO-CAT [88] range of 10 to 2060 mm by the end of the twenty-first century. We approximate this line of evidence using a uniform distribution, since CO-CAT reports no most plausible value.

As noted previously, a key aim of the study is to address how decision strategies may change based on expectations about future sea-level rise. In this capacity, the Co-CAT [88] and extended Pfeffer et al. [24] scenarios work well, since they represent two idealized sea-level rise assessments with varying upper bounds and shapes (Fig 8). The differences in the upper bounds and likelihood estimates between the two scenarios are useful reflections of the deep uncertainties surrounding potential future sea-level rise. This analysis can be refined using probabilistic sea-level rise projections and/or regional assessments for studying local impacts, which is beyond the scope of this idealized sensitivity analysis.

**Fig 9:**
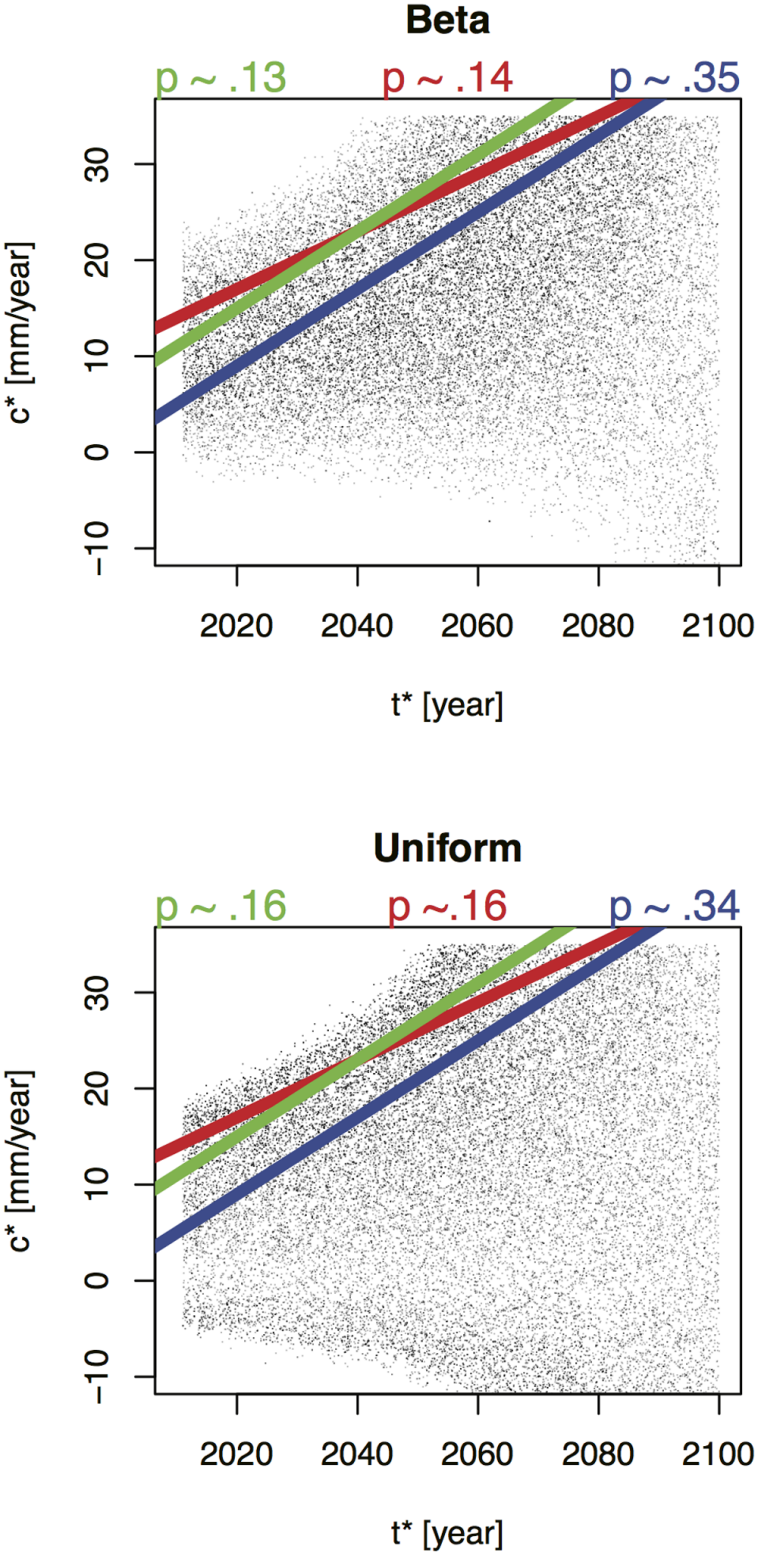
The red line shows estimates of the likelihood of the first condition describing the *Harden at Next Upgrade scenario* using the: (a) Beta distribution fit to the extended projections of Pfeffer et al. [24] and (b) the uniform distribution fit to the projections of CO-CAT [88] shown in Fig 8. Dots represent c*/t* pairs that are jointly sampled (along with polynomial parameters a, b, and c) based on the idealized sea-level rise projections (represented as beta and unform distributions). Green and blue lines show an analogous condition for two other Port of LA facilities, Berths 206–209 and Alameda and Harry Bridges Crossing, respectively.

We use the distributions derived from Pfeffer et al. [24] and CO-CAT [88] to estimate likelihoods for the condition $c^{*}\geq 14\frac{mm}{yr}+0.3\frac{mm}{yr}(t*-2010)$. As shown in Fig 9, the two distributions, though different, yield similar estimated likelihoods for this condition, of roughly 14 percent and 16 percent for the Pfeffer et al. [24] and CO-CAT [88] bounding analyses, respectively. The differences between the distirbutions and correlation between c* and t* depends on the projected sea-level rise scenario. We can express the probability that the inequality is satisfied as the narrow range:

$$14\%\leq \text{Pr}\;\left[c^{*}\geq 14\frac{mm}{yr}+0.3\frac{mm}{yr}(t^{*}-2010)\right]\leq 16\%$$ (8)

The scientific evidence regarding the condition for the hourly anomaly is even more sparse than that for c* and t*. Some studies suggest that the future hourly anomaly may remain unchanged from that currently observed. Translated into the approximation using a GEV distribution, this implies that is the distribution parameters may be assumed to be constant over time, i.e.: ψ≅517mm. For instance, the global-scale data analyses of Woodworth and Blackman [101] and Menendez and Woodworth [102] conclude that the changes in the extremes are similar to the changes in the mean. In contrast, the studies of Bromirski et al. [103] and Mendez et al. [104] find that the observed variability of sea-levels has been increasing at several locations. Cayan et al. [105] analyze model projections and place bounds on future increases in storminess near San Francisco. These bounds would correspond in our analysis to a range of 517mm ≤ ψ ≤ 533mm. (Recall that Fig 6 compares the GEV distribution with ψ = 533mm to that with ψ = 517mm).

These bounds may prove too narrow because the models used to project the short-term variability might miss important processes, such as potential changes in El Niño/Southern Oscillation properties or storm surges [82], which is why the range used in our experimental design (See Table 1) is larger. Note that the values at the high end of our experimental design range produce a storm surge of roughly 1.6 meters at the return rates of relevance to this analysis. At this time it is very difficult to define physically based bounds for future changes in storm frequency and intensity due to climate change [105]. However, future and more refined data and numerical analyses might be able to improve the estimate of the maximum surge height that different-sized storms might produce in the Port of LA, and thus develop more plausible bounding cases for the parameter ψ to be used in future analyses.

This disparate evidence regarding the likelihood of the Harden at Next Upgrade scenario can be summarized by asking the following question: Given the estimated range of likelihood for the condition on c* and t*, what range of likelihoods on the conditions for ψ and L would yield a probability for the scenario greater than its critical threshold? That is, what set of values for Pr[ψ > 533 mm] and Pr[L > 50 years], the probabilities, respectively, that ψ and L meet the conditions shown in Table 2, satisfy the equation

$$Pr\;[\psi >533mm]\cdot Pr\;[L>50\;years]\cdot \text{Pr}\;\left[c^{*}\geq 14\frac{mm}{yr}+0.3\frac{mm}{yr}(t^{*}-2010)\right]\geq 7\%$$ (9)

**Fig 10:**
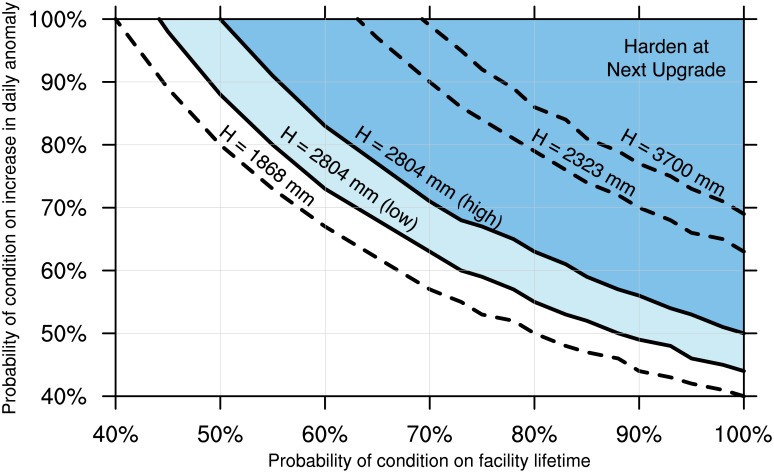
Probabilities of a long terminal lifetime (L> 50 years) and significant increase in the daily anomaly (ψ> 533 mm) required for decision to harden terminal bottoms (H = 2804 mm) (at next upgrade to pass a cost-benefit test. Dark and light shaded regions show probabilities required using high and low estimates, respectively, of likelihood of condition on c* and t*. The dashed lines show boundary of probability regions for decisions to harden at two other Port of LA facilities, Berths 206–209 and Alameda and Harry Bridges Crossing, respectively.

Fig 10 shows the resulting probability region, which suggests that Port of LA should only choose to harden its terminals at the next upgrade if it ascribes probabilities of at least about 67 percent to the conditions L>50 years and ψ>533 mm. Given that the condition on the lifetime is longer than those Port of LA has previously experienced, and the condition on the hourly anomaly increase is at the high end of available scientific evidence, Port of LA might reasonably choose not to harden at the next upgrade of this facility, even at a cost of 1 percent of the cost of the upgrade. Extended analysis of four different PoLA facilities finds one for which hardening at the next upgrade might prove warranted [106].

## 5. Comparision with a full probabilistic analysis

It proves useful to compare this Robust Decision Making analysis with a full probabilistic analysis of the same Port of LA decision considered above. Table 3 summarizes some of the key differences between Robust Decision Making and a probabilistic analysis, using three attributes: how uncertainties are characterized, the decision criteria used, and the decision process with decision makers [107].

**Table 3:** Comparison of RDM and Probabilistic Risk Analysis (PRA) methods.

	Probabilistic Risk Analysis (PRA)	Robust Decision Making (RDM)
Characterization of uncertainty	Well-characterized (single joint probability distributions)	Deep uncertainty—Characterize vulnerabilities of proposed strategies
Decision criteria	Optimal Choice	Robustness (satisfice over wide range of futures)
Decision process	Experts gather evidence and provide rankings to decision makers	Deliberation with analysis

The probabilistic analysis uses the best available science to estimate a single joint probability distribution for all the uncertain input parameters in Table 1. The decision model would use probability density functions for all the inputs, rather than distinguishing between well-characterized and deeply uncertain factors. As its decision criterion, the probabilistic analysis seeks the optimal strategy for the best-estimate distribution, rather than a strategy robust over a wide range of futures. Finally, the probabilistic analysis envisions that experts gather information and provide a rankings of strategies to decision makers, while the Robust Decision Making analysis envisions a process of deliberation with analysis in which analysts and decision makers work together to propose strategies and understand their vulnerabilities.

The right-most column of Table 1 shows our best estimate probability distribution for each of the model input parameters. The distributions of the model parameters were produced by fitting to both the observational data and the extended Pfeffer and CO-CAT scenarios. These fits also provide distributions for the a, b, c, c*, and t* parameters. The correlations between these later parameters and c* and t* are weak (Fig 9). The Robust Decision Making analysis in this study ignores them, while the full probabilistic analysis includes them. We have no information to distinguish the relative likelihood of the Pfeffer et al. and CO-CAT scenarios, so we assume a uniform prior—that is, each are assumed to be equally likely.

We use a set of GEV distributions, with the scale spanning the full range, 517mm ≤ ψ ≤ 543mm, described in the literature. With no information to distinguish the relative likelihood of these values, a uniform prior is assumed. As in the Robust Decision Making analysis, the location of hourly anomaly distribution for value of ψ is given by Eq (6) to leave the mean annual sea-level unchanged. We have no information to determine the relative likelihood of different values of the maximum allowable overtop probability, p_crit_, so we assume a uniform prior over the range used in the Robust Decision Making analysis. We also lack any information to determine the relative likelihood of different values of the terminal lifetime. But many analyses do show the results of full probabilistic analyses as a function of a range of values for a single parameter; a convention we will adopt here.

**Fig 11:**
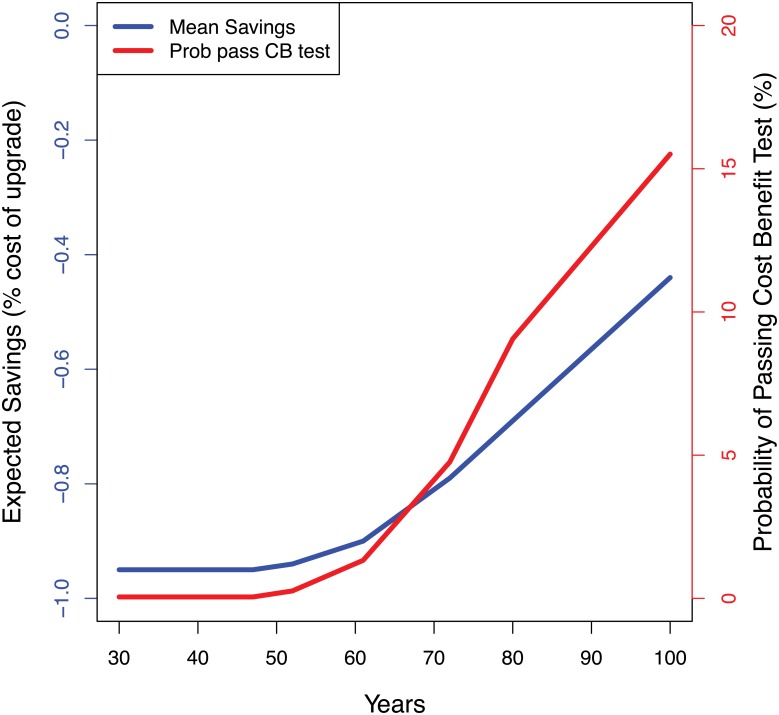
Results of full probabilistic analysis showing expected cost of hardening at next upgrade and probability of passing a cost-benefit test as a function of the terminal lifetime.

Fig 11 shows the results of this full probabilistic analysis, plotting the probability that hardening at the next upgrade would pass a cost-benefit test and the expected cost as a function of the terminal lifetime L. These results use 1954 Monte Carlo samples for each value of L. The probability of a positive cost-benefit is never high—at most, 16 percent for terminal lifetimes of 100 years. Not until the terminal lifetime exceeds about 50 years do our calculations show a greater than 1 percent probability that early hardening passes a cost-benefit test. Note an important distinction between the samples based on the Robust Decision Making analysis and the full probabilistic analysis: In the Robust Decision Making-analysis, the quasi-random Latin Hypercube sample aims only to explore the full range of plausible model results. The sample makes no statement about the relative likelihood of alternative cases in the real world. In the full probabilistic analysis, the Monte Carlo sample aims to reproduce our best-estimate of the likelihood of cases in the real world, in order to facilitate the calculation of expected cost.

The Robust Decision Making and full probabilistic analyses thus appear to give similar recommendations. Both suggest that Port of LA should not harden its terminals at the next upgrade at a cost of 1 percent (or higher) of the total upgrade cost. Only in situations where the terminal lifetime is very long, greater than about 50 years, is there any possibility that such hardening would pass a cost-benefit test, and even in such cases the probability that it would do so seems low.

As the decision support literature makes clear, however, a final policy prescription is not the only, nor even primary, relevant attribute of a decision support methodology [11]. In particular, the transparency and credibility of the information provided to decision makers, and the process by which they interact with the information can prove at least as important. In this light, it is important to note that the Robust Decision Making and PRA analyses provide different information to decision makers and envision two different types of engagement with stakeholders. As shown in Table 3, the full probabilistic analysis collects all the analysts’ judgments at the start of the process. Once the probability distributions over future states of the world are defined, the analysis yields recommendations that follow deductively from the probability estimates and the simple, but explicit, representations of the decision makers’ preferences (e.g., the adopted decision-criterion of an expected benefit-cost ratio). As its primary products, the analysis provides distributions of the outputs of interest to decision makers—in this case, the expected cost of an early upgrade, and a ranking of the desirability of alternative decisions. In this case, the early upgrade is less desirable because its expected cost exceeds that of not upgrading. Sensitivity analysis can also suggest which uncertain input parameters contribute most to the variance of the outputs.

The Robust Decision Making analysis follows a more complicated process and one that employs analysts’ and decision makers’ judgments at more stages. The process begins by focusing on a specific proposed decision. Analysts then create an experimental design over the uncertain model input parameters designed to test this decision, judging which uncertainties to treat as well characterized and which to treat as deep. Next analysts and decision makers use simulation model results to identify scenarios where the policy fails to meet its goals—in this case, where a decision to harden at the next upgrade fails a cost-benefit test. Analysts then present the scientific evidence that could help decision makers decide whether such scenarios are sufficiently likely to justify taking an alternative decision. The Robust Decision Making analysis does not in general produce a ranking of strategies, but rather provides information to help decision makers weigh their choices. As part of this process, the Robust Decision Making analysis explicitly describes the scenarios where a proposed policy may fail to meet its goals and defines a probability threshold—that is, the likelihood that a decision maker would ascribe to that scenario in order to justify taking action to address it.

The two approaches also embody different treatments of uncertainty, which we can usefully summarize with reference IPCC uncertainty guidance. This guidance provides a template for judging confidence in scientific judgments based on the level of supporting evidence and agreement [108].

**Fig 12:**
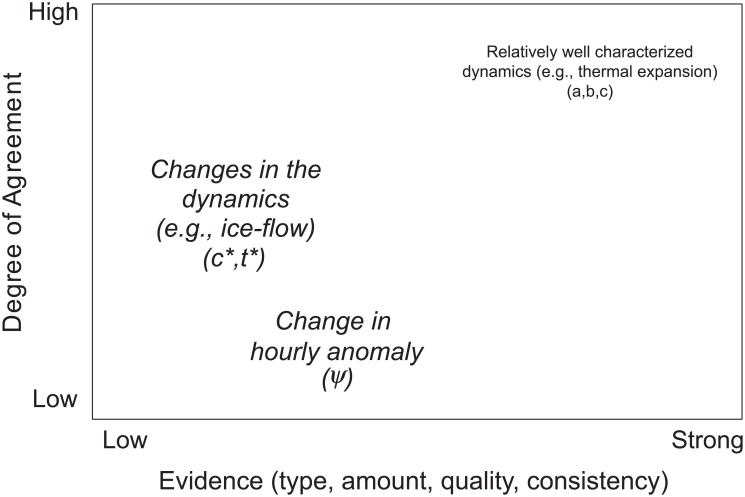
Assessment of the evidence and level of agreement underlying the scientific information used in this analysis, following the characterization method of Mastrandrea et al. [108]. The size of the text reflects the importance of information to Port of LA’s decision. Italics show that the factor was considered deeply uncertain in the Robust Decision Making analysis.

Fig 12 uses this template from the IPCC uncertainty guidance [108] to summarize the scientific information about future sea-level rise used in our Robust Decision Making and full probabilistic analyses. The plot (Fig 12) shows the a, b, and c parameters in the upper right-hand corner because, as described above, there exists a high level of both evidence and agreement that the polynomial model structure fit to past observations should provide reasonable projections of the contributions of future sea-level rise due to well-resolved processes such as thermal expansion. If these terms were more properly represented as a mix of well and less well-understood processes, the former would remain in this upper right-hand corner and the latter would reside elsewhere on the figure. The figure shows the c* and t* in the middle left-hand side, because there is little direct observational evidence for potential changes in the system dynamics (for example by “rapid dynamical changes in ice-flow” [22]). However, there exists some agreement on the upper bounds on such contributions to sea-level rise over the next century (Fig 1). Fig 12 shows ψ in the lower middle left because few studies and little agreement exist on how climate change might affect the future hourly anomaly at Port of LA, but the worldwide diversity of current storm surge patterns in different locations with different topographies may provide useful evidence for estimating upper bounds on what the port might expect over the twenty-first century.

The results in Section 4, as would an analysis of variance in the full probabilistic analysis, makes clear that Port of LA’s decision whether or not to harden at the next upgrade depends strongly on scientific estimates in which we have low confidence. The full probabilistic analysis does not distinguish between varying levels of uncertainty when informing investment decisions. This distinction suggests that the standard probabilistic approach can tend to lead to overconfident results, because it undervalues the importance of low-confidence information. The Robust Decision Making analysis, in contrast, distinguishes between information with different levels of confidence, differentiating between relatively well-characterized uncertainty (e.g., sea-level rise that follows the past observed dynamics) and deeply uncertain information (e.g., abrupt potential future dynamic changes of land ice, the hourly anomaly, or the characteristics of future terminal management). Thus, a decision maker using a standard probabilistic approach may be more vulnerable to low confidence information than the decision maker using the Robust Decision Making approach.

The full probabilistic analysis embodies a concept where experts assemble the best available science so that this information can be used to inform a ranking of alternative decision options. However, as noted previously, this approach can lead to underestimation of risk in the presence of deep uncertainty, since it does not distinguish between differing levels of scientific confidence [51]. The Robust Decision Making analysis embodies a scenario concept that explicitly distinguishes among differing levels of scientific confidence. In addition, the analysis helps decision makers recognize potentially stressing cases, consider how they might respond, and communicate this information within and externally to their organization. In other work, we have described how decision analytic approaches such as Robust Decision Making that begin with policies, identify vulnerabilities, and then suggest potential responses can lead to more productive engagement with decision makers than do approaches that rank options based on specified probabilities [48,99,109]. Here we show how the former approach can also represent in the analysis information with differing levels of uncertainty, which may also facilitate engagement with decision makers.

## 6. Discussion and conclusions

This study presents a robust decision making framework for addressing potential investment decisions under deep uncertainty. Proposed investment decisions at the Port of LA are analyzed as a traceable application of the methods. Results show how an organization such as the Port of LA can evaluate the potential for presumably low probability but large impact levels of extreme future sea-level rise in its infrastructure investment decisions. Considering such extreme climate changes can prove difficult because of the deep uncertainty involved, not only in any scientific projections, but also regarding any expectations of future socioeconomic conditions that may affect judgments about the value of alternative near-term infrastructure investments.

This study addresses this challenge by employing Robust Decision Making to address the following question: Should Port of LA make an additional investment to harden its facilities against potential extreme future sea-level rise during the next major upgrade of those facilities? Robust Decision Making represents one of a number of new decision analytic approaches that address deep uncertainty by beginning with a specific set of options facing a decision maker and then identifying specific information about the uncertain future that might affect the decision makers’ choice among those options. The approach used here is based on two questions: (1) Under what future conditions would a Port of LA decision to harden its facilities at the next upgrade pass a cost-benefit test, and (2) Does current science and other available information suggest that such conditions are sufficiently likely to justify such an investment?

In particular, this study’s Robust Decision Making analysis evaluates the benefits and costs of a Port of LA decision to harden at the next upgrade over 500 cases representing a wide range of assumptions about future sea-level rise and its future facility management. The analysis next uses a cluster analysis on the resulting database of simulation model outcomes to concisely describe scenarios where a decision to harden passes a cost-benefit test and estimates a probably threshold for those scenarios, that is the likelihood Port of LA would need to ascribe to the scenario to choose to harden. Finally, the analysis evaluates the scientific evidence that would suggest whether the scenario is sufficiently likely or unlikely to justify a decision to harden.

Our analysis employs many simplified representations of important physical uncertainties and processes, which introduce important caveats and also point to future research. For example, the sea-level projections do not include the effects of different greenhouse gas forcing projections [82,110]. We do not consider regional variations in sea-level projections from the global mean [38]. The simple sea-level rise model fails to account for the complex mixture of response time-scales [29,61,111,112]. We assume that future changes in the dynamics of the system (e.g., due to changes in ice-flow dynamics) introduce a step-function change in the rate of sea-level rise, when in reality such changes would happen over time. In particular, some fraction of past sea-level changes are due to changes in land-ice and model hindcasts and projections of this component are deeply uncertain [113–116]. In representing all past sea-level rise as well-characterized uncertainty, we neglect considerable structural uncertainties about the most appropriate mixture of functional forms [117]. The Robust Decision Making framework used in this study could accommodate these richer physical descriptions and their multiple levels of attendant uncertainties. For instance, these processes might be represented with more complex models that resolve more of the relevant processes using physically motivated parameterizations [29,86]. These more complex models would introduce additional uncertain parameters into the Robust Decision Making analysis. While the neglect of such processes in this study would not seem to affect any of our conclusions, including them in more detailed treatments of port infrastructure investment decisions would make for useful further research.

We apply Robust Decision Making to a single proposed future investment decision, but the methodologies could be broadened to increase the robustness of the decision. For example, recent work using a route-map approach suggests that early implementation of low-regrets measures in advance of a proposed decision time point can increase the robustness of adaptation decisions related to sea-level changes [65]. Such measures could include: strengthening existing defenses, implementing a monitoring framework, and continued research into the causes and effects of changes in extreme sea-level rise events. In particular, monitoring and research would likely lead to improved understanding of key physical processes controlling sea-level changes, such as melting land ice and changes in storminess.

This study also compares its Robust Decision Making analysis of a decision to harden the terminal bottoms to a full probabilistic analysis. Such an analysis uses the best available science to estimate a single joint probability distribution for the uncertain model input parameters and then calculates the expected savings from an investment to harden and the probability that such an investment passes the cost-benefit test.

The Robust Decision Making and full probabilistic analyses give similar recommendations to the Port of LA regarding the investment considered here. It is important to note, however, that the two approaches differ in several key aspects. First, the full probabilistic provides decision makers with the expected savings from an investment, based on the best scientific estimates. The Robust Decision Making analysis begins by describing the conditions where such an investment would pass a cost-benefit test, estimates the probability such a scenario would have to have to justify making the investment, and then assembles the scientific evidence that can help a decision maker judge whether or not the investment is worthwhile. In some cases, Robust Decision Making can provide important additional understanding about the problem for decision-makers and analysts. For example, the case example highlighted here shows how the Robust Decision Making approach identifies decision-relevant uncertainties as well as the location of undesirable outcomes in a high-dimensional parameter space. This is an important insight because it points to areas where a refined analysis and future research has the potential to improve the decision. Robust Decision Making can also better represent important properties of the decision-problem compared to the probabilistic approach. Previous studies have shown that tools used in Robust Decision Making can result in considerably different strategies than the probabilistic approach when deep uncertainty is large and the decision-maker cares about deeply uncertain outcomes [12,13,118]. In situations where decision makers have confidence in the best scientific estimates of the probability distributions, the full probabilistic analysis provides a more streamlined approach. But in situations, such as those faced by Port of LA as it considers the potential for extreme sea-level rise, where the scientific estimates of probabilities are at best imprecise, approaches such as Robust Decision Making may provide a more convenient and transparent framework for organizing the relevant scientific information and applying it to the decision.

Robust Decision Making has several advantages over full probabilistic analysis, including the ability to provide additional insights for decision makers and to enable a representation of deep uncertainty. These advantages come with costs. Robust Decision Making generally requires more computation. In this example, this extra computation was trivial, involving 500 evaluations of a simple benefit-cost excel spreadsheet requiring only a few minutes on a standard laptop. In some examples, however, the extra computation could prove substantial. In addition, a Robust Decision Making analysis has more steps than PRA, some of which require human intervention and judgment. While such interventions can prove a major benefit when the analytics are used facilitate deliberation among decision makers, it may prove a drawback in other decision contexts. Finally, this Robust Decision Making analysis required more detailed queries of the climate information. While the probabilistic analysis used only the probability density functions, the Robust Decision Making analysis also required judgments about the confidence in various probability density functions and for those regarded as deeply uncertain, the extent to which the weight of the evidence lies above or below the critical thresholds for the harden at next upgrade decision. In general, Robust Decision Making can be interpreted as an expansion of the analytical tools needed for an expected utility analysis that neglects aspects such as deep uncertainty. There are, of course, situations where an expected utility analysis under risk can be appropriate and the added complexities of Robust Decision Making can be avoided. As shown above, the problem discussed in this study is not one of these cases. Many problems involving climate change fall into the same category [10,118].

## Supporting information

S1 Table Details and sources to the numbers in Fig 1. (DOCX)

S1 File Compressed folder containing data, source code and plot routines for reproducing the essential results. (ZIP)
