# Supplementary material for: Characterizing uncertain sea-level rise projections to support investment decisions
Source: PLoS One. 2018 Feb 7;13(2):e0190641. doi: 10.1371/journal.pone.0190641 (PMC5802450; doi:10.1371/journal.pone.0190641)
Supplement: S1 Table — (DOCX) [file pone.0190641.s001.docx]

| **Name** | **Highest projection in 2100 (cm)** | **Emission Scenario Assumed** | **Year (relative to year)** | **Source** |
| --- | --- | --- | --- | --- |
| IPCC FAR | 110 | IPCC90A | 2100 (1990) | Warrick and Oerlemans 1990 (Figure 9.6, p 277) |
| IPCC SAR | 86 | IS92a | 2100 (1990) | Warrick et al 1996 (Table 7.8, p 381) |
| IPCC TAR | 88 | A1FI | 2100 (1990) | Church et al 2001 (p 642) |
| IPCC AR4 | 59 | A1FI | 2090–2099 (1980-1999) | Meehl et al 2007 (Table 10.7. p 820) |
| IPCC AR5 | 98 | RCP8.5 | 2100 (1986–2005) | Church et al 2013 (p 1186) |
| Pfeffer et al 2008 | 201 | N/A | 2100 | Pfeffer et al 2008 (p 1342) |
| Vermeer and Rahmstorf 2009 | 179 | A1FI | 2100 (1990) | Vermeer and Rahmstorf 2009 (p 21531) |
| Lowe et al 2009 | 250 | N/A | 2095 (1990) | Lowe et al 2009 (p 32) |
| Church et al 2011 | 80 | A1FI | 2100 (1980-1999) | Church et al 2011 (p 133) |
| Katsman et al 2011 | 115 | A1FI | 2100 (1990) | Katsman et al 2011 (p 631) |
| Sriver et al 2012 | 225 | RCP8.5 | 2100 | Sriver et al 2012 (p 898) |
| Parris et al 2012 | 200 | N/A | 2100 (1992) | Parris et al 2012 (Table 2, p 12) |
| NRC 2012 | 140 | A1FI | 2100 (2000) | National Research Council 2012 (Table 5.2, p 89) |
| Jevrejeva et al 2012 | 165 | RCP8.5 | 2100 (1980-2000) | Jevrejeva et al 2012 (Table 3, p 17) |
| Miller et al 2013 | 270 | RCP8.5 | 2100 (2000) | Miller et al 2013 (p 7) |
| Horton et al 2013 | 150 | RCP8.5 | 2100 (2000) | Horton et al 2014 (Table 1, p 3) |
| Jevrejeva et al 2014 | 180 | RCP8.5 | 2100 (2000) | Jevrejeva et al 2014 (p 4) |
| Kopp et al 2014 | 245 | RCP8.5 | 2100 (2000) | Kopp et al 2014 (p 389) |
| Kopp et al 2016 | 131 | RCP8.5 | 2100 (2000) | Kopp et al 2016 (p E1437) |
| Sweet et al 2017 | 250 | RCP8.5 | 2100 (2000) | Sweet et al 2017 (p 22) |
| Le Bars et al 2017 | 292 | RCP8.5 | 2100 (1987-2005) | Le Bars et al 2017 (p 4) |
