# Supplementary figures and images for: Characterizing uncertain sea-level rise projections to support investment decisions
Source: PLoS One. 2018 Feb 7;13(2):e0190641. doi: 10.1371/journal.pone.0190641 (PMC5802450; doi:10.1371/journal.pone.0190641)

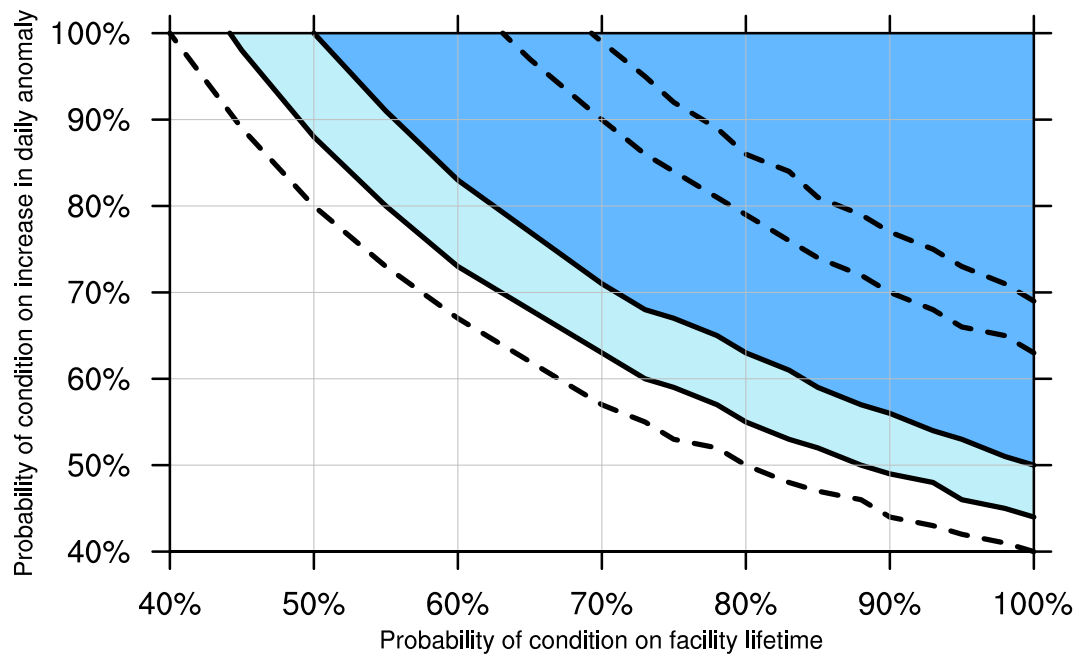

Supplement: S1 File — (ZIP) [file pone.0190641.s002.zip › pola_package_2017.12.02/rdm_analysis/Figure_10/test.pdf]

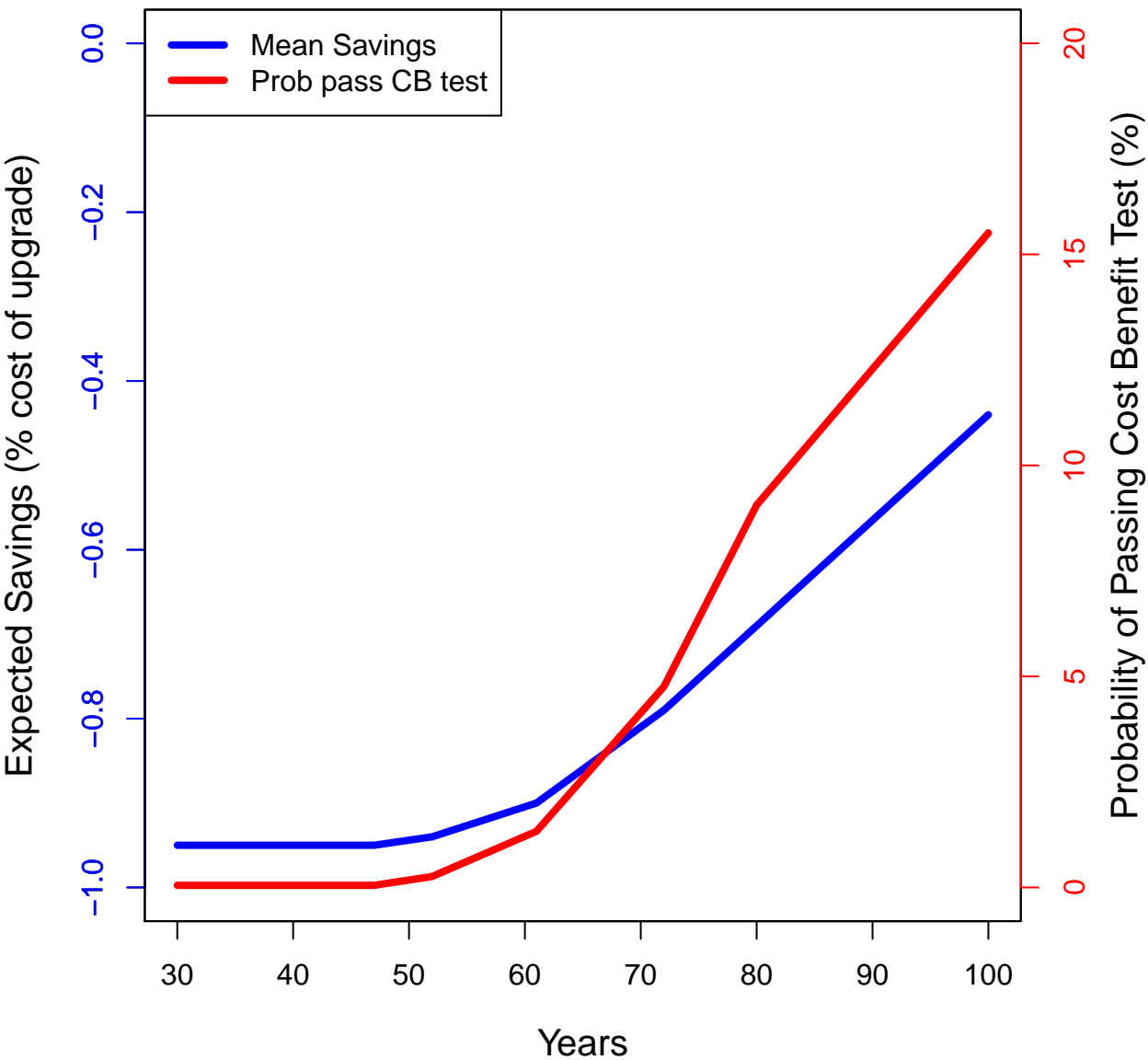

Supplement: S1 File — (ZIP) [file pone.0190641.s002.zip › pola_package_2017.12.02/rdm_analysis/pra_figure/figure_11_R.pdf]

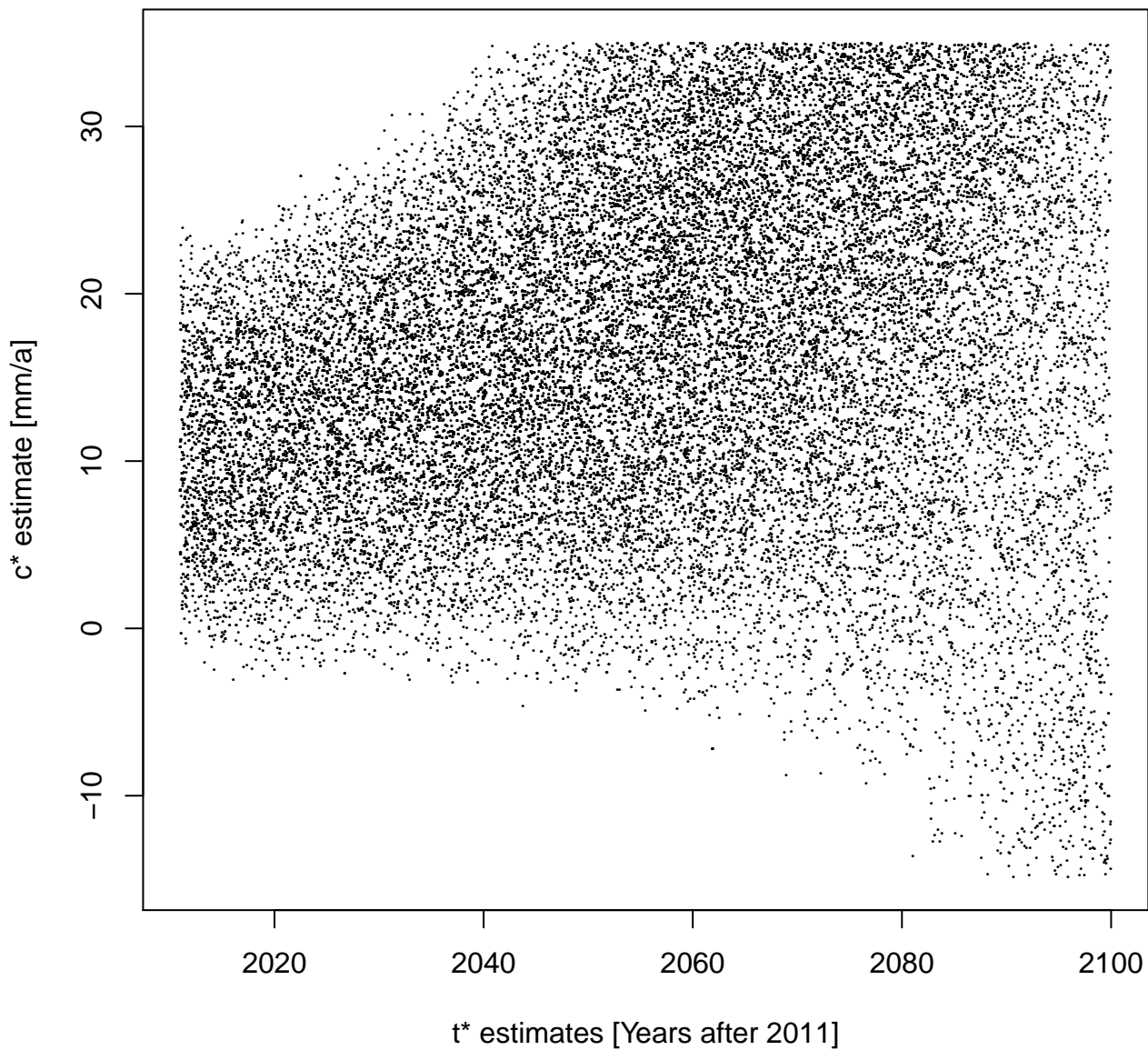

Supplement: S1 File — (ZIP) [file pone.0190641.s002.zip › pola_package_2017.12.02/slr_analysis/beta/output/c*vst*.pdf]

**(a) Extended Scenario of Pfeffer et al (2008)**

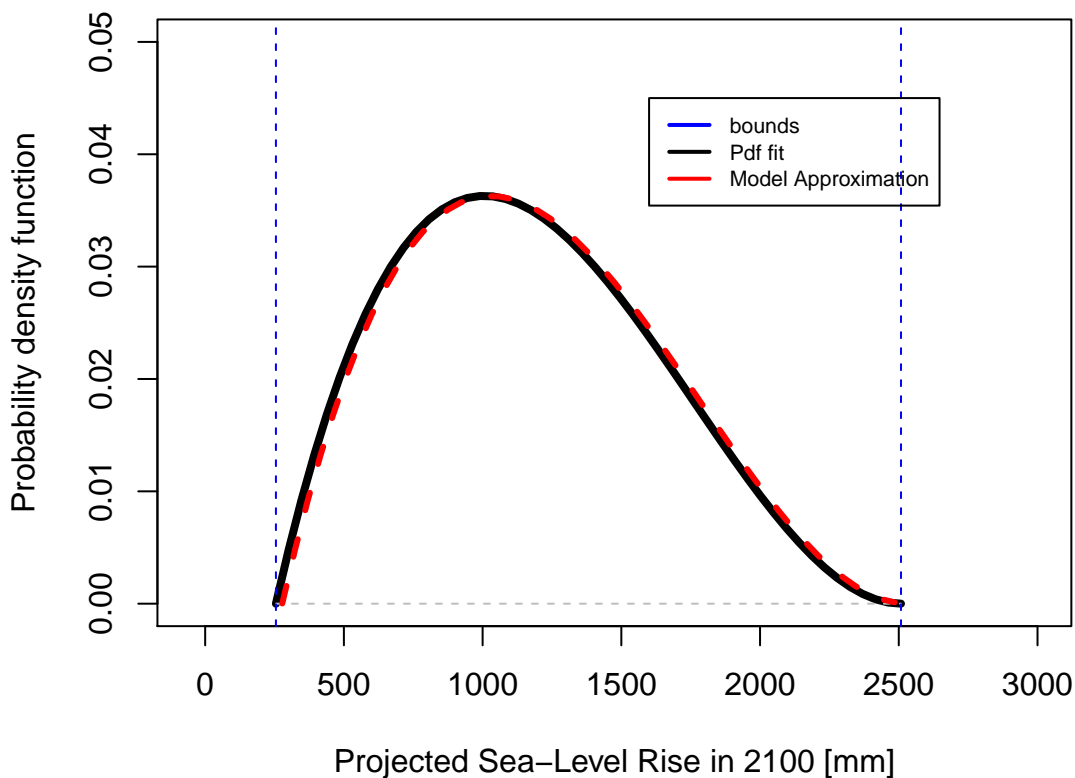

**(b) Extended Scenario of Co-CAT (2010)**

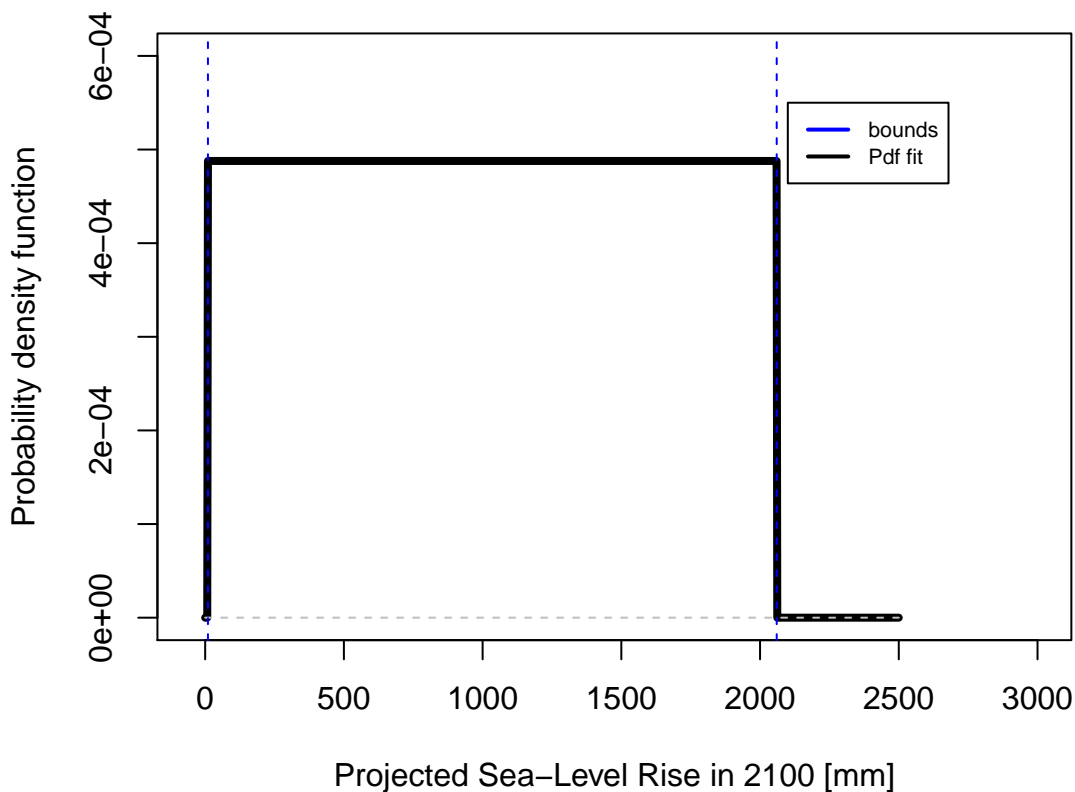

Supplement: S1 File — (ZIP) [file pone.0190641.s002.zip › pola_package_2017.12.02/slr_analysis/beta/output/fitted_beta.pdf]

mean sea-level anomaly (mm) with respect to the year 2000

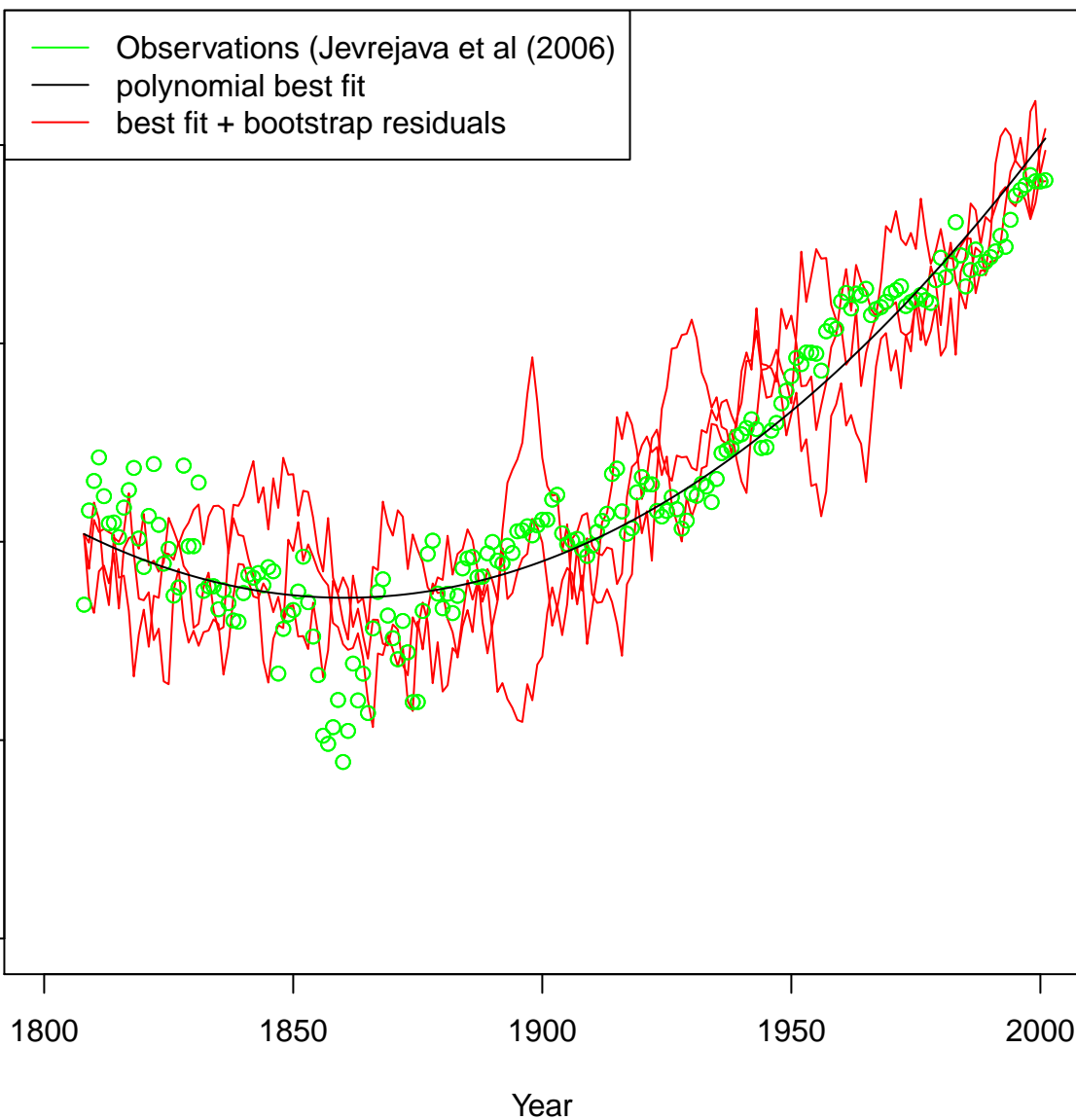

Supplement: S1 File — (ZIP) [file pone.0190641.s002.zip › pola_package_2017.12.02/slr_analysis/beta/output/hindcast.pdf]

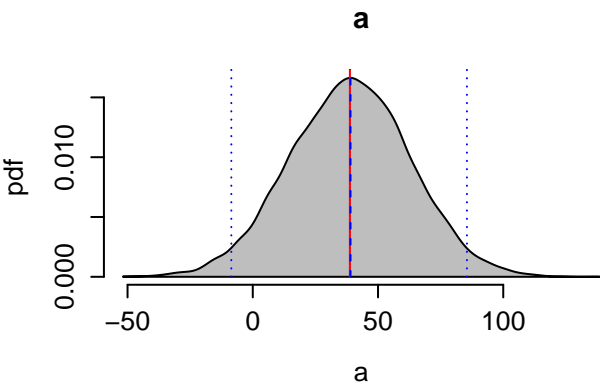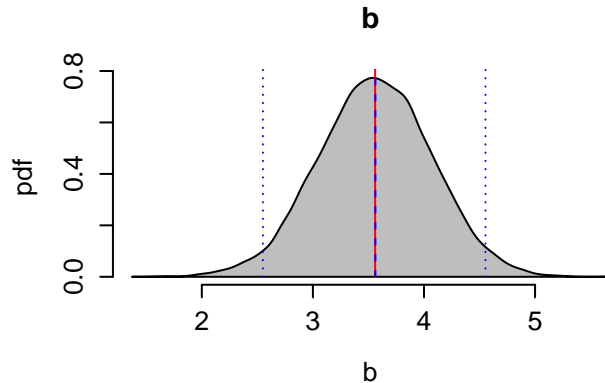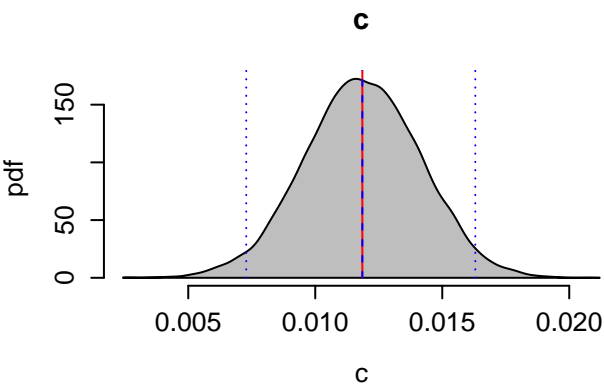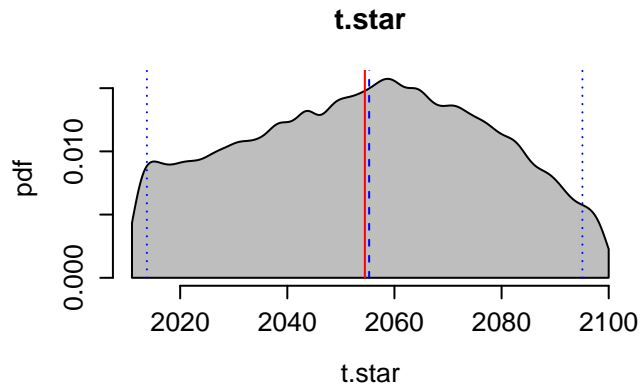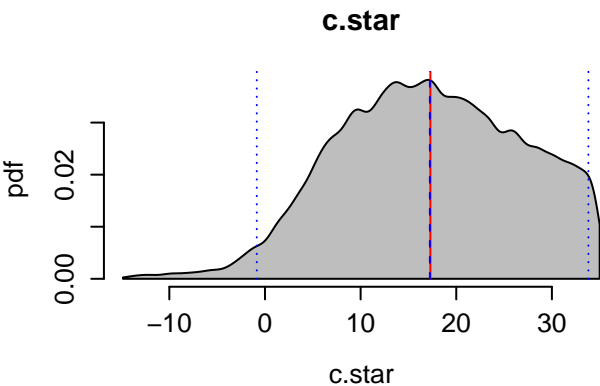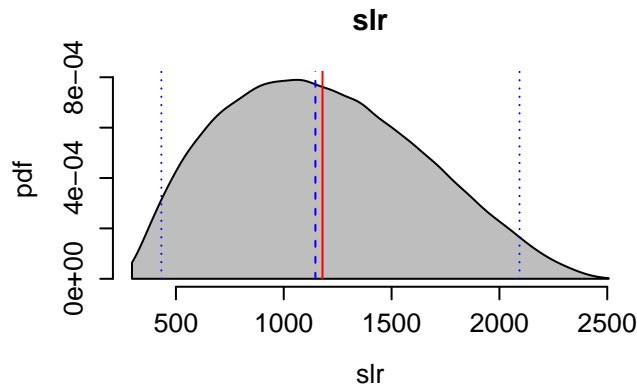

Supplement: S1 File — (ZIP) [file pone.0190641.s002.zip › pola_package_2017.12.02/slr_analysis/beta/output/marginals.pdf]

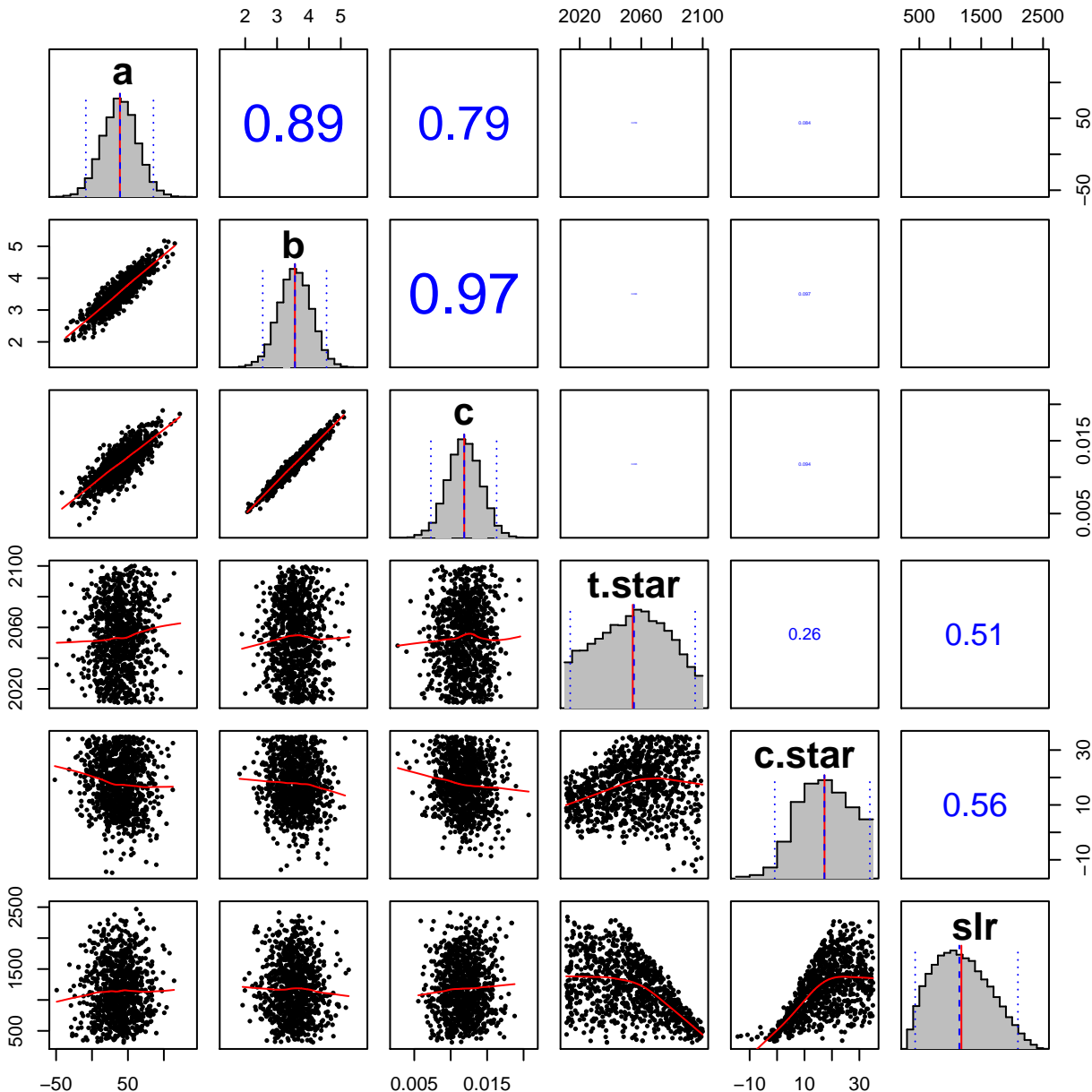

Supplement: S1 File — (ZIP) [file pone.0190641.s002.zip › pola_package_2017.12.02/slr_analysis/beta/output/pairs.pdf]

mean sea-level anomaly (mm) with respect to the year 2000

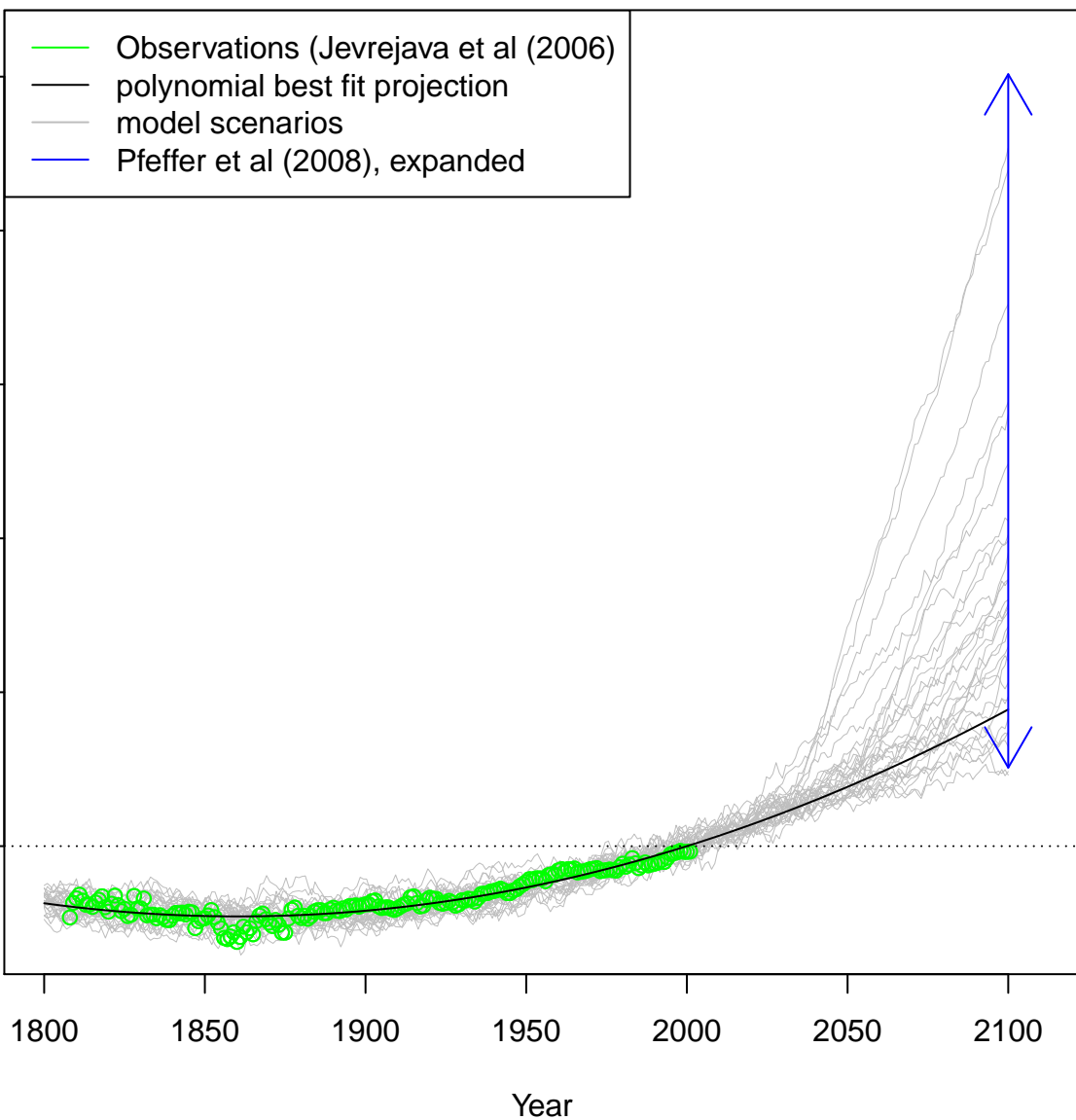

Supplement: S1 File — (ZIP) [file pone.0190641.s002.zip › pola_package_2017.12.02/slr_analysis/beta/output/projections.pdf]

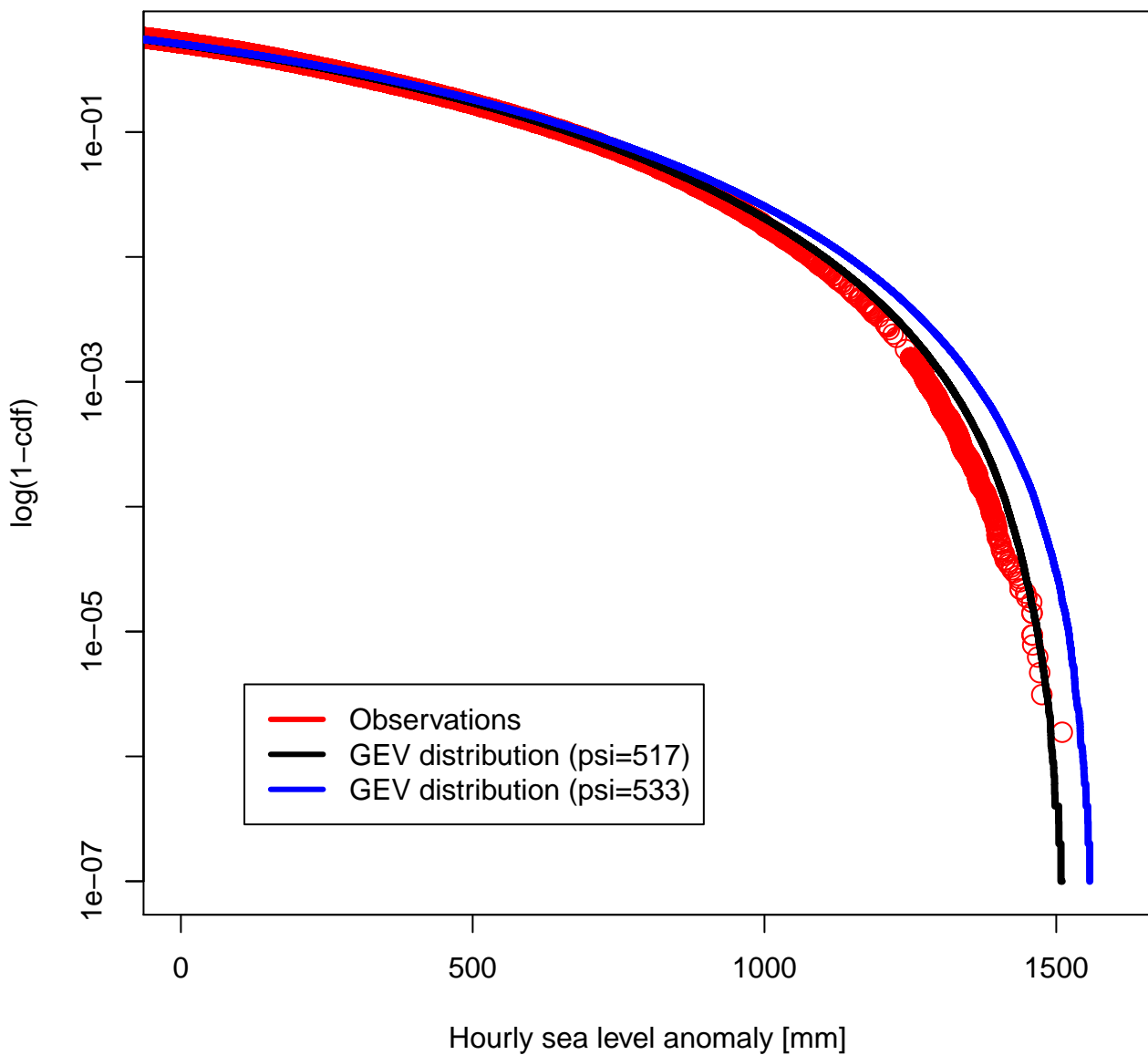

Supplement: S1 File — (ZIP) [file pone.0190641.s002.zip › pola_package_2017.12.02/slr_analysis/Figure_6_tail/figure.pdf]

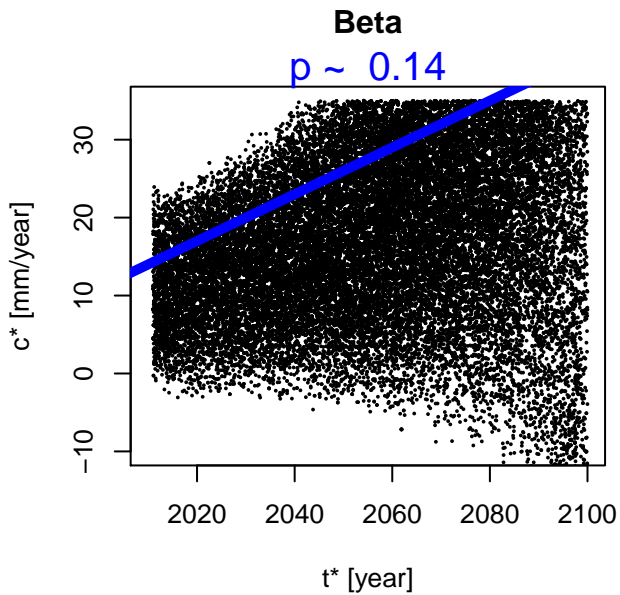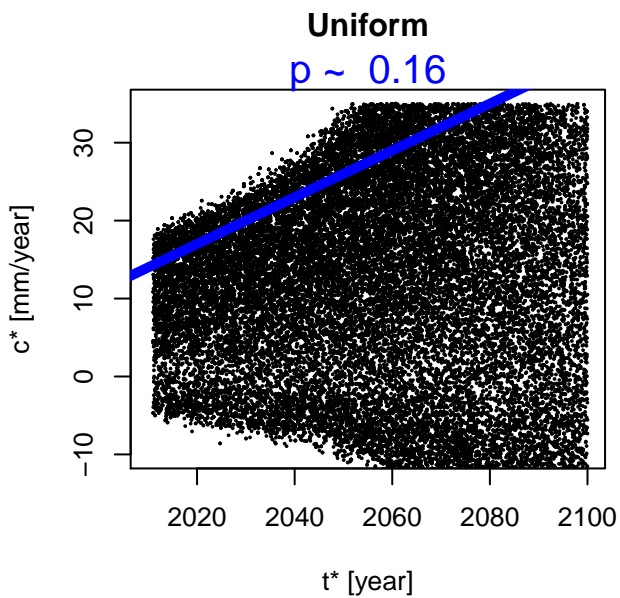

Supplement: S1 File — (ZIP) [file pone.0190641.s002.zip › pola_package_2017.12.02/slr_analysis/p_boxes/plots/p_box_0.3_14.pdf]

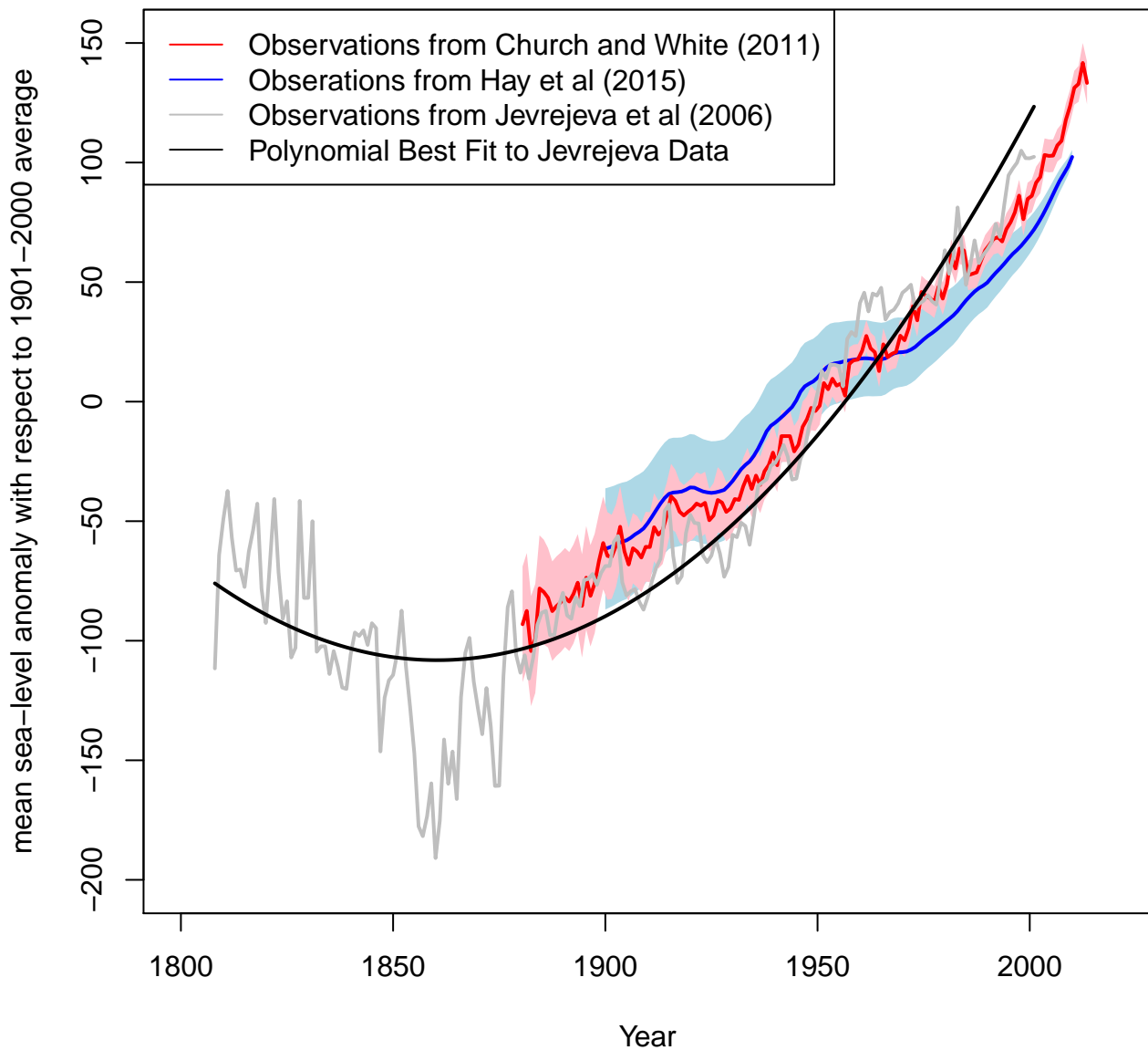

Supplement: S1 File — (ZIP) [file pone.0190641.s002.zip › pola_package_2017.12.02/slr_analysis/slr_obs_plot/slr_time_series.pdf]

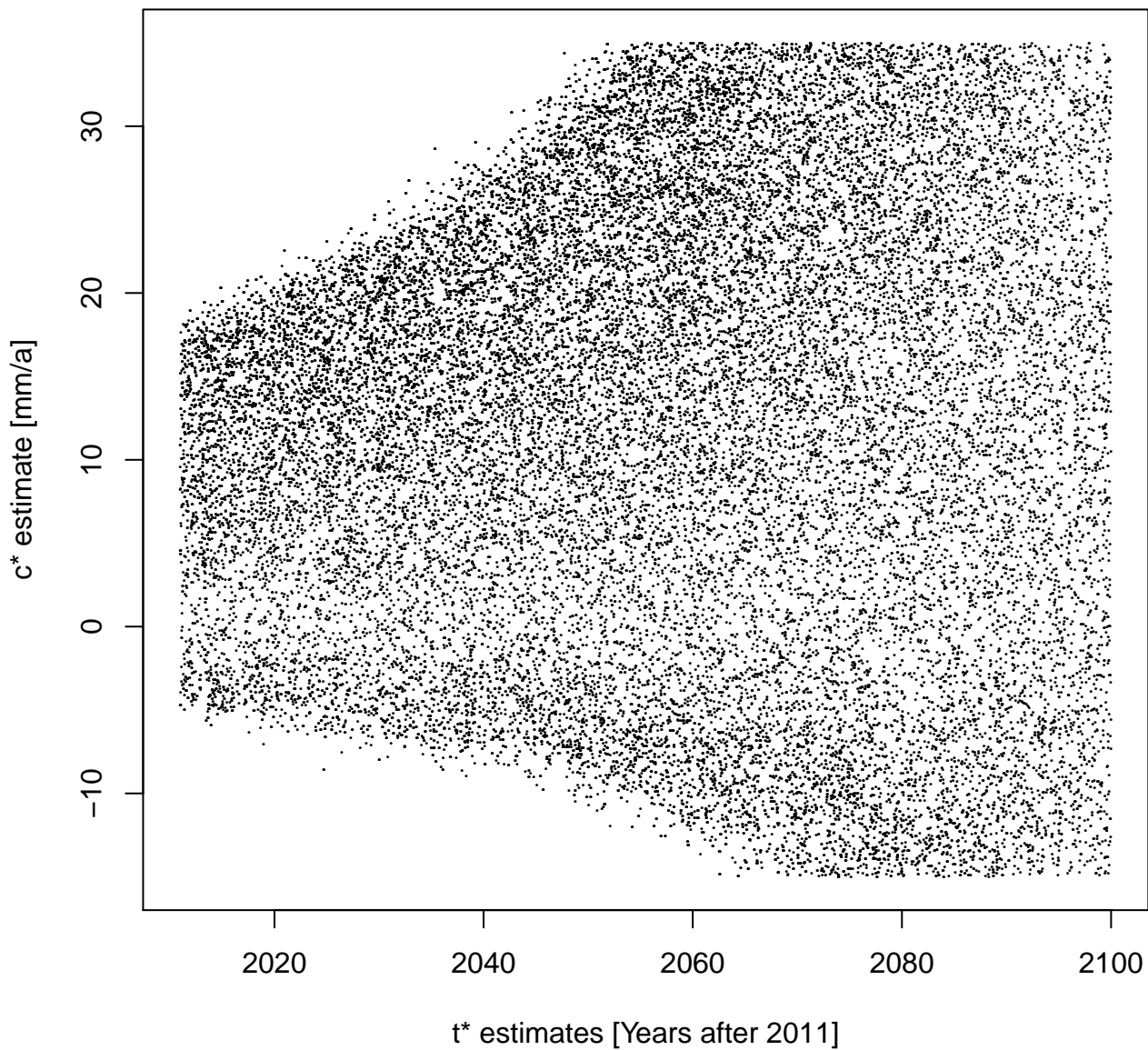

Supplement: S1 File — (ZIP) [file pone.0190641.s002.zip › pola_package_2017.12.02/slr_analysis/uniform/output/c*vst*.pdf]

**(a) Uniform Distribution**

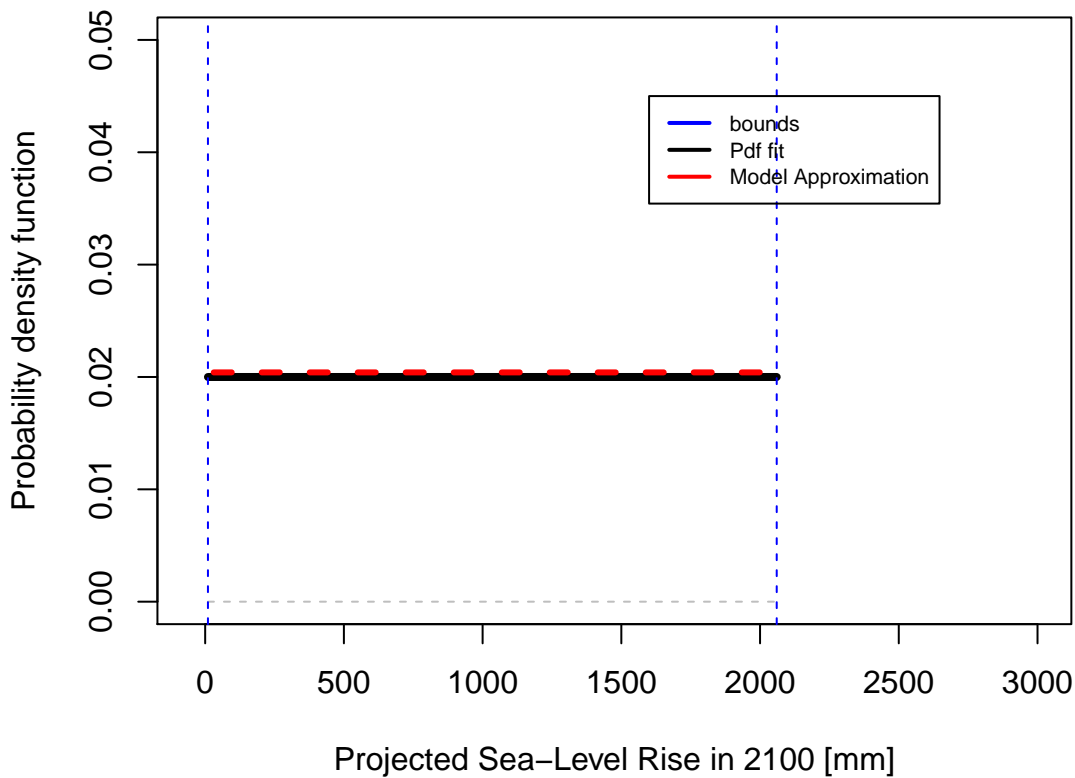

**(b) Extended Scenario of Co-CAT (2010)**

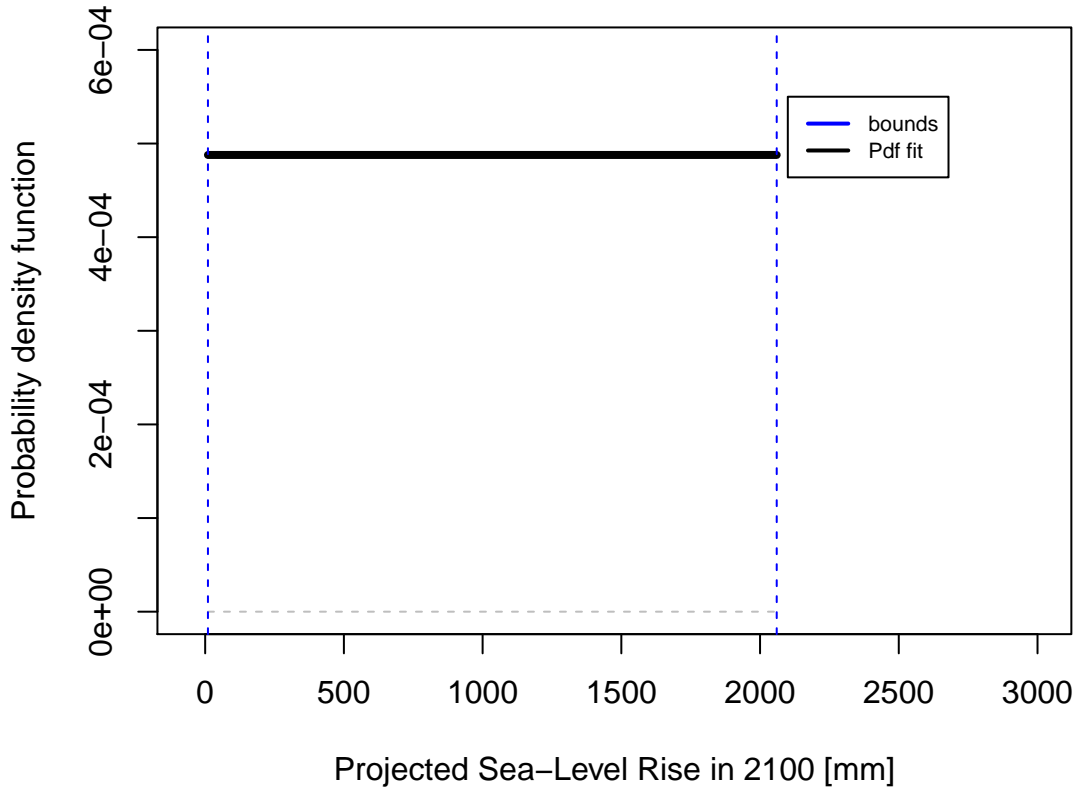

Supplement: S1 File — (ZIP) [file pone.0190641.s002.zip › pola_package_2017.12.02/slr_analysis/uniform/output/fitted_uniform.pdf]

mean sea-level anomaly (mm) with respect to the year 2000

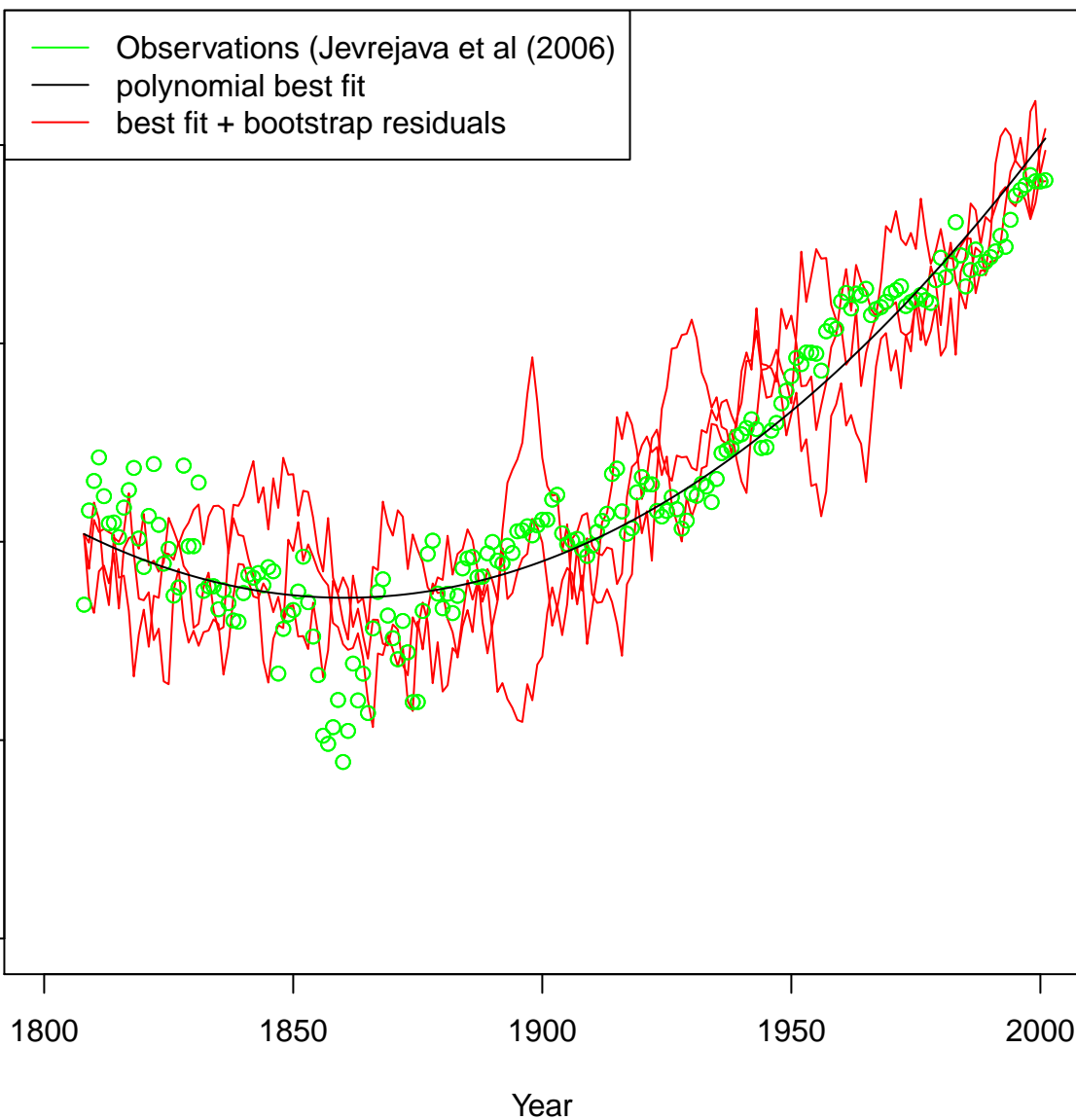

Supplement: S1 File — (ZIP) [file pone.0190641.s002.zip › pola_package_2017.12.02/slr_analysis/uniform/output/hindcast.pdf]

mean sea-level anomaly (mm) with respect to the year 2000

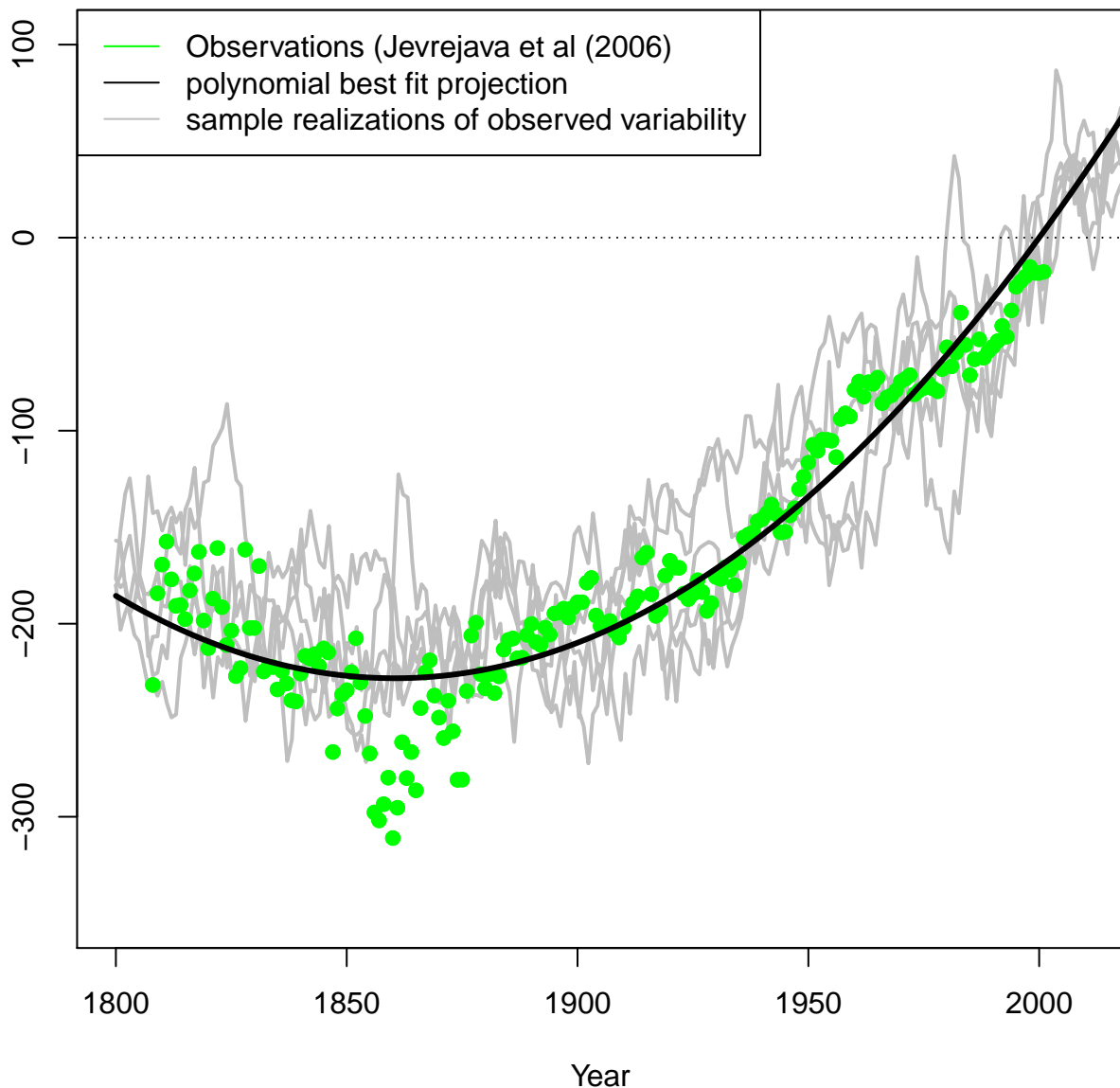

Supplement: S1 File — (ZIP) [file pone.0190641.s002.zip › pola_package_2017.12.02/slr_analysis/uniform/output/hindcast_format.pdf]

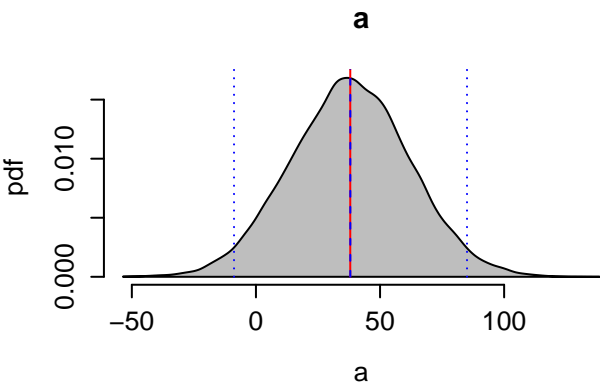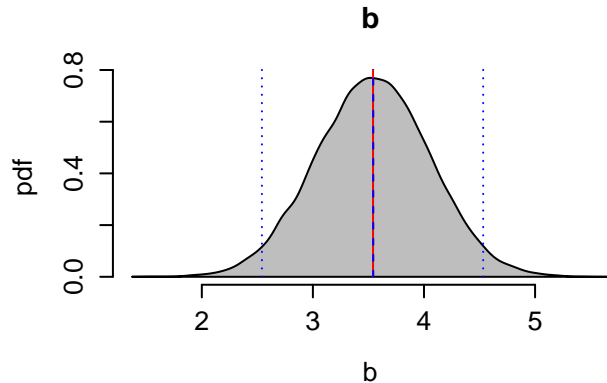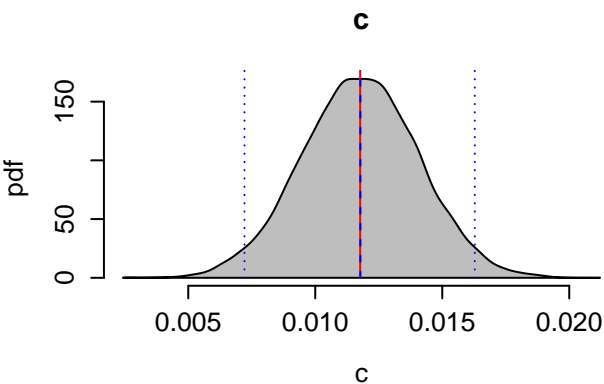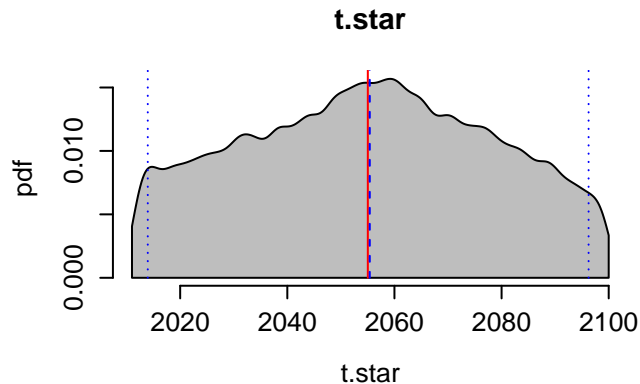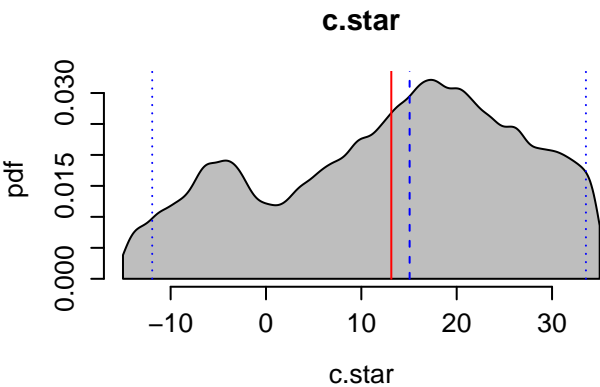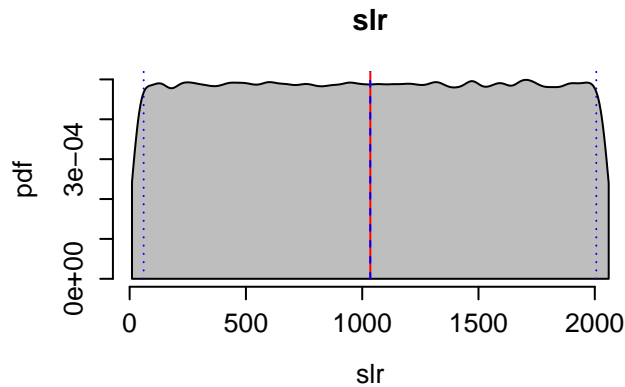

Supplement: S1 File — (ZIP) [file pone.0190641.s002.zip › pola_package_2017.12.02/slr_analysis/uniform/output/marginals.pdf]

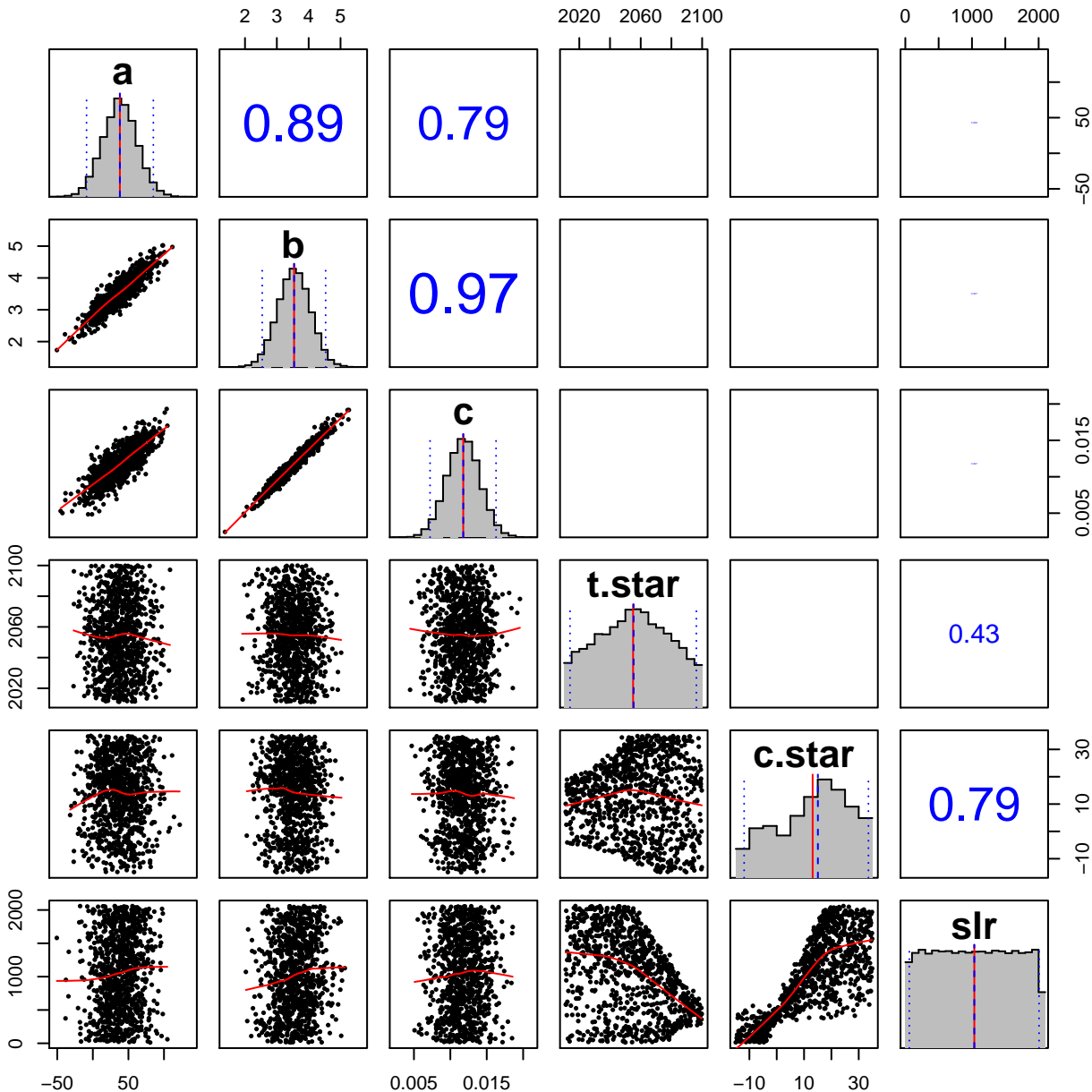

Supplement: S1 File — (ZIP) [file pone.0190641.s002.zip › pola_package_2017.12.02/slr_analysis/uniform/output/pairs.pdf]

mean sea-level anomaly (mm) with respect to the year 2000

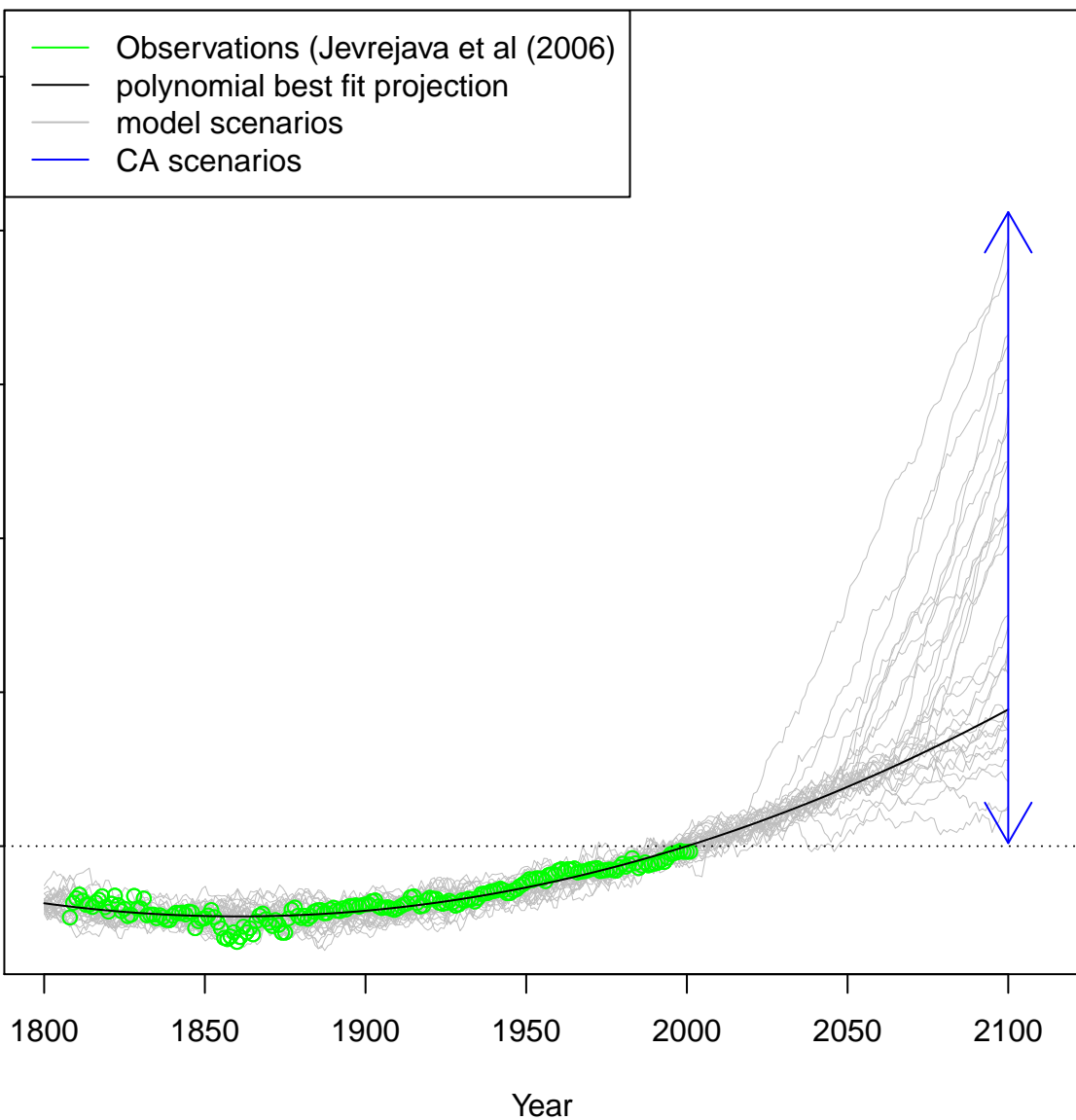

Supplement: S1 File — (ZIP) [file pone.0190641.s002.zip › pola_package_2017.12.02/slr_analysis/uniform/output/projections.pdf]
